# Transposable elements are the primary source of novelty in primate gene regulation

**DOI:** 10.1101/gr.218149.116

**Published:** 2017-10

**Authors:** Marco Trizzino, YoSon Park, Marcia Holsbach-Beltrame, Katherine Aracena, Katelyn Mika, Minal Caliskan, George H. Perry, Vincent J. Lynch, Christopher D. Brown

**Affiliations:** 1 Department of Genetics, Perelman School of Medicine, University of Pennsylvania, Philadelphia, Pennsylvania 19104, USA;; 2 Institute for Biomedical Informatics, University of Pennsylvania, Philadelphia, Pennsylvania 19104, USA;; 3 Department of Human Genetics, University of Chicago, Chicago, Illinois 60637, USA;; 4 Departments of Anthropology and Biology, Pennsylvania State University, University Park, Pennsylvania 16802, USA

## Abstract

Gene regulation shapes the evolution of phenotypic diversity. We investigated the evolution of liver promoters and enhancers in six primate species using ChIP-seq (H3K27ac and H3K4me1) to profile *cis*-regulatory elements (CREs) and using RNA-seq to characterize gene expression in the same individuals. To quantify regulatory divergence, we compared CRE activity across species by testing differential ChIP-seq read depths directly measured for orthologous sequences. We show that the primate regulatory landscape is largely conserved across the lineage, with 63% of the tested human liver CREs showing similar activity across species. Conserved CRE function is associated with sequence conservation, proximity to coding genes, cell-type specificity, and transcription factor binding. Newly evolved CREs are enriched in immune response and neurodevelopmental functions. We further demonstrate that conserved CREs bind master regulators, suggesting that while CREs contribute to species adaptation to the environment, core functions remain intact. Newly evolved CREs are enriched in young transposable elements (TEs), including Long-Terminal-Repeats (LTRs) and SINE-VNTR-*Alu*s (SVAs), that significantly affect gene expression. Conversely, only 16% of conserved CREs overlap TEs. We tested the *cis*-regulatory activity of 69 TE subfamilies by luciferase reporter assays, spanning all major TE classes, and showed that 95.6% of tested TEs can function as either transcriptional activators or repressors. In conclusion, we demonstrated the critical role of TEs in primate gene regulation and illustrated potential mechanisms underlying evolutionary divergence among the primate species through the noncoding genome.

The contribution of *cis*-regulatory elements (CREs) to phenotypic and behavioral evolution has been shown in many taxa (King and Wilson 1975; Prabhakar et al. 2008; Schmidt et al. 2010; Cain et al. 2011; Marnetto et al. 2014; Zhou et al. 2014; Prescott et al. 2015; Reilly et al. 2015; Villar et al. 2015; Emera et al. 2016; Berthelot et al. 2017). Although previous studies have suggested a role for transposable elements (TEs) in the evolution of gene regulation, validating the functional contribution of TEs in mammalian gene regulation remains a challenge (McClintock 1950, 1984; Britten and Davidson 1969; Davidson and Britten 1979; Jordan et al. 2003; Bejerano et al. 2006; Wang et al. 2007; Bourque et al. 2008; Sasaki et al. 2008; Markljung et al. 2009; Kunarso et al. 2010; Lynch et al. 2011, 2015; Schmidt et al. 2012; Chuong et al. 2013, 2016; Jacques et al. 2013; Xie et al. 2013; del Rosario et al. 2014; Sundaram et al. 2014; Du et al. 2016; Rayan et al. 2016; Ward et al. 2017).

A significant fraction of the accessible regions in primate genomes overlap a TE (Jacques et al. 2013). Similarly, the recruitment of novel regulatory networks in the uterus was likely mediated by ancient mammalian TEs (Lynch et al. 2011, 2015). Conversely, neocortical enhancers do not exhibit strong evidence of transposon co-option (Emera et al. 2016).

Many important questions remain unanswered: To what extent are regulatory elements functionally conserved across primates? Are specific genomic features predictive of CRE conservation? To what extent have TEs driven the evolution of gene regulation?

To address these questions, we investigated the evolution of gene expression and regulation in the primate liver. While liver functions are largely conserved across primates, different environmental exposures, diets, and lifestyles shape the adaptation of liver functions, making this tissue a suitable model in which to explore the conservation and divergence of gene regulation.

We performed ChIP-seq for histone H3 lysine 27 acetylation (H3K27ac) and histone H3 lysine 4 monomethylation (H3K4me1), which mark functional and poised regulatory elements, in the liver of six primate species, including at least one species from each major primate clade (Perelman et al. 2011). We generated RNA-seq data from the same specimens and estimated the degree of evolutionary conservation of regulatory activity and gene expression levels across the entire lineage. We identified genomic features associated with the evolutionary conservation of gene regulation. Finally, to functionally characterize the contribution of TEs to gene expression divergence, we performed extensive experimental validation on TE-derived CREs.

## Results

### Data generation, quality assessment, and validation

**Figure 1.**
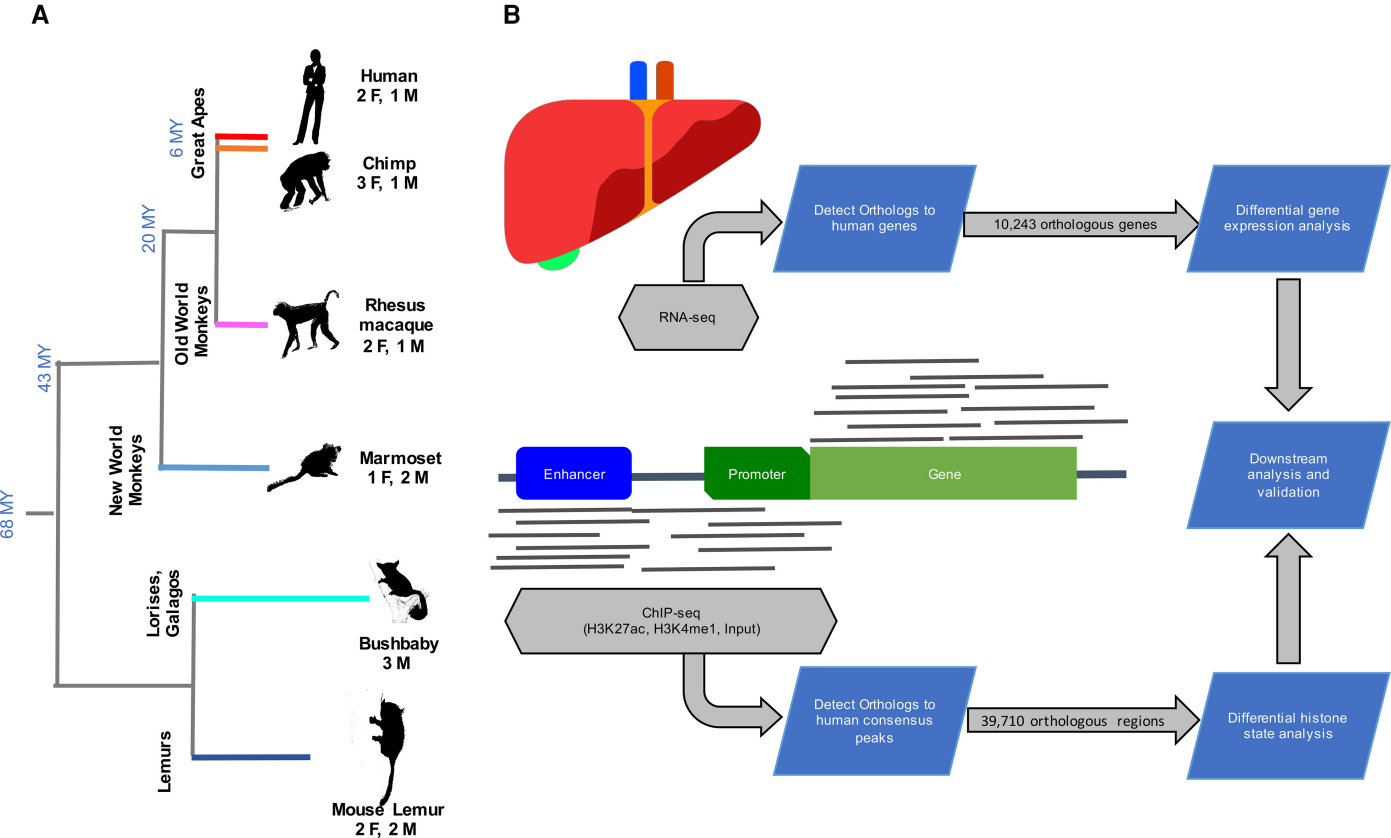
Experimental design and analytical pipeline. (*A*) Sampling included three to four specimens from six species representing all major primate clades. (*B*) ChIP-seq and RNA-seq profiles were produced from the liver samples. Differential histone modification and gene expression analyses were performed on the orthologous CREs and genes in each species, respectively.

We generated RNA-seq and ChIP-seq data from post mortem livers of three or four individuals per species of mouse lemur (*Microcebus murinus*), bushbaby (*Otolemur garnettii*), marmoset (*Callithrix jacchus*), rhesus macaque (*Macaca mulatta*), chimpanzee (*Pan troglodytes*), and human (*Homo sapiens*) (Fig. 1). The samples were from young adults and, with the exception of the bushbaby, included both males and females. After stringent quality control, a total of 18 RNA-seq and 14 ChIP-seq samples remained post quality control (QC) and were used for the analyses (Supplemental Table S1). We identified H3K27ac and H3K4me1 peaks in the human liver, treating all human individuals as replicates in the peak calling procedure with MACS2 (FDR < 5%) (Zhang et al. 2008). Overlapping peaks from the two histone marks were merged.

Several lines of evidence indicate that the regions of histone modification we have identified represent active CREs. First, 66.1% of ENCODE HepG2 H3K27ac regions overlapped one of our human peaks (The ENCODE Project Consortium 2012). Moreover, 71.6% of the 244,269 Roadmap liver H3K27ac regions and 49.7% of the 233,386 Roadmap liver H3K4me1 regions overlapped one of our human peaks (Roadmap Epigenomics Mapping Consortium 2015). The vast majority of predicted CREs did not exhibit significant sex-biased regulatory activity (Supplemental Table S8). Finally, we tested the regulatory activity of 1-kb fragments from 276 predicted CREs in HepG2 cells using a novel parallelized reporter assay (Supplemental File S4; Melnikov et al. 2012; Patwardhan et al. 2012; Sharon et al. 2012): 191 drove significant reporter activity (69.2%) (Supplemental Table S6), demonstrating that the majority of the CREs predicted from our ChIP-seq data are likely functional regulatory elements in the human liver.

### The majority of human six-way-alignable CREs are functionally conserved across primates

We identified regions orthologous to human peaks from the genomes of nonhuman primates using the Ensembl multiple sequence alignment (MSA) database (Flicek et al. 2014; Yates et al. 2015). We cataloged, excluding the sex chromosomes, 39,710 total human CREs with orthologs in all six species: 32,759 enhancers (distance from nearest transcription start site [TSS] > 1 kb) and 6951 promoters (distance from nearest TSS < 1 kb).

After extracting ChIP-seq read counts for the six species from the 39,710 regions, we assessed evidence of differential histone modification with DESeq2 (Love et al. 2014), using ChIP-seq input data as a covariate. We compared the read counts across all possible human-centric species × species and group × group pairwise comparisons. This approach provides a quantitative assessment of histone modification profiles across species, while avoiding issues arising from experimental variables that may confound peak calling (Waszak et al. 2015). An analysis of human and marmoset, the latter being the species with the smallest number of peaks called in this study, strongly supported the robustness of this approach (for detailed analysis, see Supplemental File S4; Supplemental Fig. S1). Additionally, we compared our H3K27ac data to a recent study of liver CREs in mammals (Villar et al. 2015). Only 10.9% of human H3K27ac peaks at FDR < 5% (5.8% with FDR < 1%) exhibited evidence of differential histone modification between two studies (Supplemental File S4; Supplemental Fig. S7).

**Figure 2.**
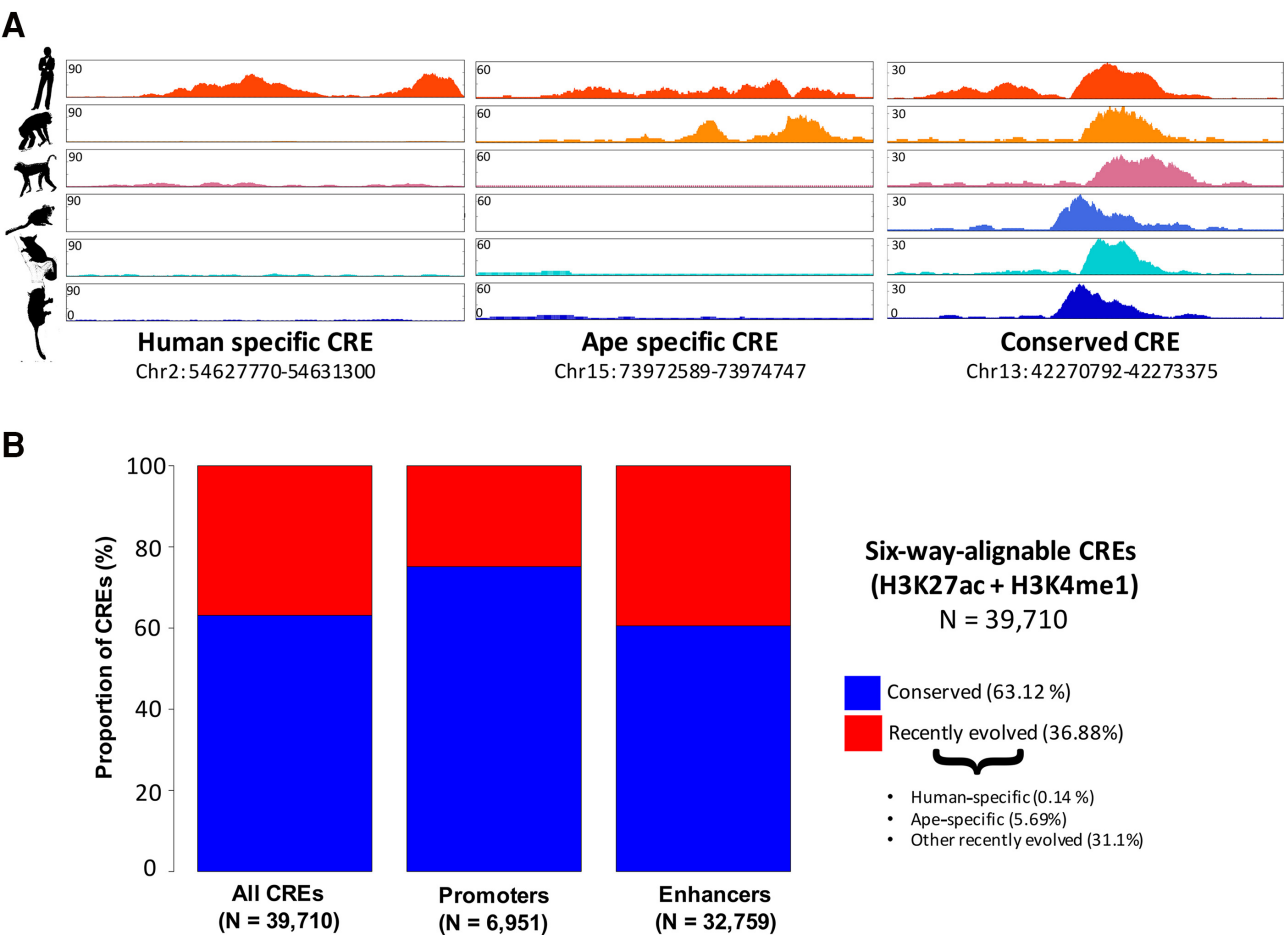
Primates CREs are evolutionarily conserved. (*A*) Examples of human-specific, ape-specific, and conserved CREs. (*B*) Fraction of conserved and recently evolved primate CREs, with breakdown of enhancers and promoters.

The majority of the 39,710 human CREs (25,067 CREs [63.1%]; FDR < 10%) did not exhibit significant differential histone modification in any of the tested pairwise comparisons (Fig. 2). This suggests that these regulatory regions are consistently active across the primate lineage and thus may represent evolutionarily conserved primate CREs. This conclusion was robust to changes to the FDR threshold (Supplemental Table S7). As an additional control, we performed a chimpanzee-centric analysis for regions orthologous to chimpanzee H3K27ac consensus peaks and demonstrated that 54.1% of these regions were not differentially histone modified in any of the pairwise comparisons, indicating that species-specific bias is unlikely.

**Figure 3.**
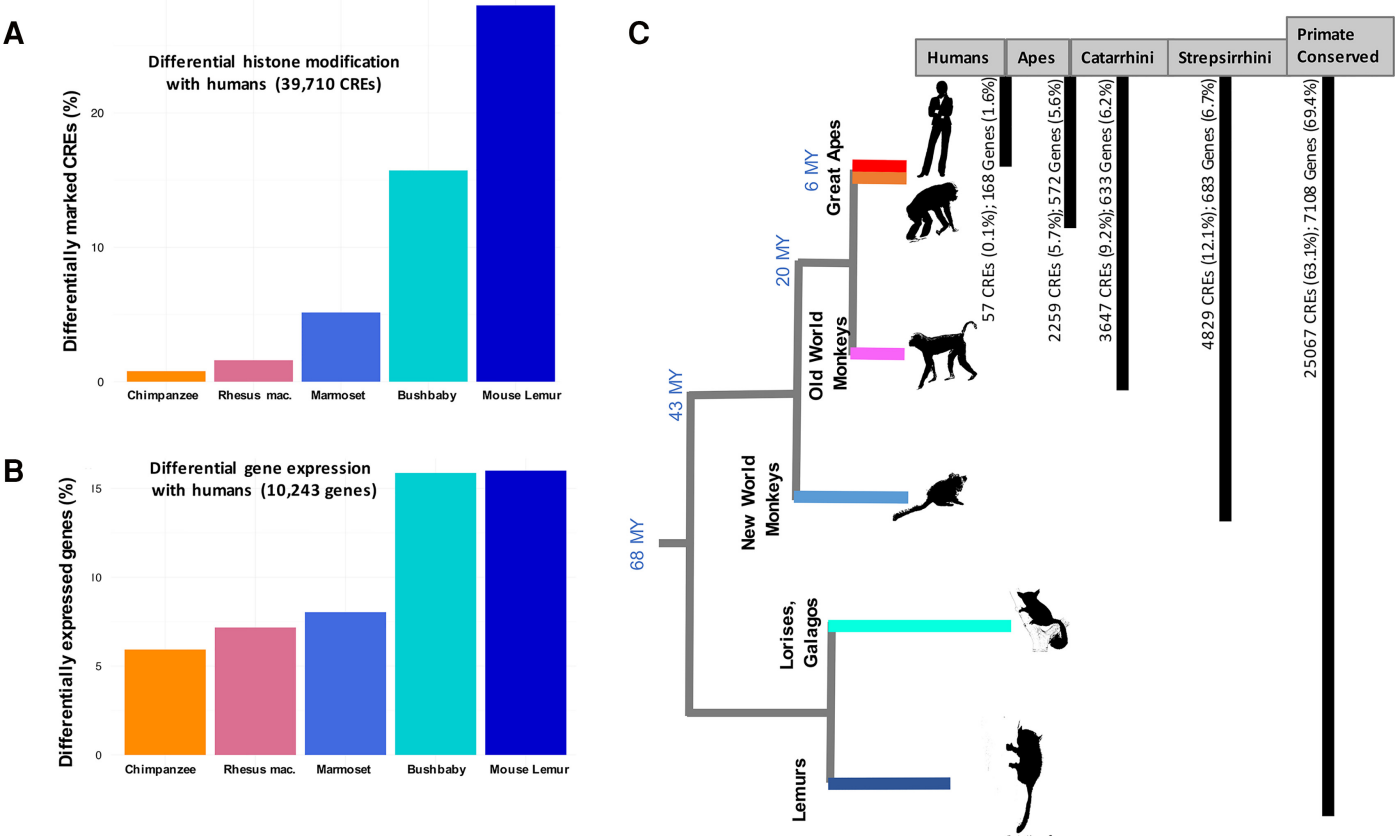
Differential histone mark and gene expression across species. (*A*) Human-centric pairwise comparisons for differential histone modification states on 39,710 orthologous CREs. (*B*) Human-centric pairwise comparisons for differential gene expression of 10,243 orthologous genes. (*C*) Number of lineage-specific CREs and genes across the primate phylogeny.

Primate promoters were more conserved than enhancers (75.1% and 60.5%, respectively; Fisher's exact test *P* < 2.2 × 10^−16^) (Fig. 2), as observed previously in mammals (Villar et al. 2015). On the other hand, 36.9% of orthologous CREs exhibited differential histone modification state across species (Fig. 2). We detected 57 human-specific CREs (0.14%) and 2259 ape-specific CREs (5.7%) (Fig. 3; Supplemental Table S2). Together, our differential histone state analysis results are broadly supported by several studies that have consistently suggested a high degree of regulatory element conservation between closely related species in metazoans (Cotney et al. 2013; Boyle et al. 2014; Prescott et al. 2015; Emera et al. 2016). We note that the estimated fraction of conserved CRE is lower (36.2%: 39.6% of promoters and 24.3% of enhancers) when analyses are not restricted to six-way orthologous regions (i.e., treating all human CREs lacking an ortholog in any of the other species as not conserved). However, we remark that some primate genome assemblies, particularly mouse lemur and bushbaby, are largely incomplete, and the lack of orthology is most likely a consequence of assembly quality. We thus limited our analyses to the six-way-alignable CREs.

To assess the extent to which restricting this analysis to the set of six-way-orthologous CREs could affect our results, we re-performed the same analysis for H3K27ac, only considering human, chimpanzee, and rhesus macaque. Of human H3K27ac CREs, 86.9% have an aligned ortholog in both species. In total, 98.3% and 97.5% of the tested CREs were conserved between human and chimpanzee (99.0% in the six-species analysis) and between human and rhesus macaque (98.6% in the six-species analysis), respectively. Results between the two analyses are thus comparable (Fisher's exact test *P* > 0.05 for both of the comparisons), suggesting that the restriction of our analyses to six-way orthologous CREs did not significantly bias the results.

### Conservation of the nucleotide sequence is associated with conservation of regulatory activity

For each human-centric species × species comparison, we estimated the following: (1) the fraction of differentially modified CREs, (2) the fraction of differentially expressed genes from a set of 10,243 genes with six-way orthologs (Supplemental Table S3), and (3) the per-nucleotide pairwise sequence divergence for each species with respect to humans for each of the 39,710 unique orthologous CREs.

Differential histone modification ranged from 0.79% in the human × chimpanzee to 28.4% in the human × mouse lemur comparisons (Fig. 3A). Similarly, differential gene expression ranged from 5.93% in the human × chimpanzee to 16.0% in the human × mouse lemur comparisons (Fig. 3B). Both differential histone modification and differential gene expression reflected phylogenetic distance between humans and other tested species, and differentially expressed genes were significantly more likely to be associated with a differentially modified CRE than expected by chance (Cochran–Mantel–Haenszel test odds ratio [OR] = 1.45; *P* < 2.2 × 10^−16^).

Sequence divergence was significantly associated with differential histone modification (human × chimpanzee, logistic regression *P* = 6.8 × 10^−8^; human × rhesus macaque, *P* < 2.2 × 10^−16^; human × marmoset, *P* = 1.3 × 10^−8^; human × bushbaby, *P* = 3.3 × 10^−4^; human × mouse lemur, *P* = 2.4 × 10^−4^) (Supplemental Table S9). Moreover, functionally conserved CREs were significantly more likely to overlap a placental mammal phastCons element (Siepel et al. 2005) than expected by chance (Fisher's exact test *P* < 2.2 × 10^−16^). Together, these data demonstrate that CREs with conserved nucleotide sequence are significantly more likely to have conserved regulatory activity and are associated with conserved gene expression, as previously suggested (Brown et al. 2007; Cooper and Brown 2008; Pollard et al. 2010; Gittelman et al. 2015; Yang et al. 2015; Dong et al. 2016; Holloway et al. 2016; Lewis et al. 2016).

### Genomic features associated with CRE conservation and rapid evolution

**Figure 4.**
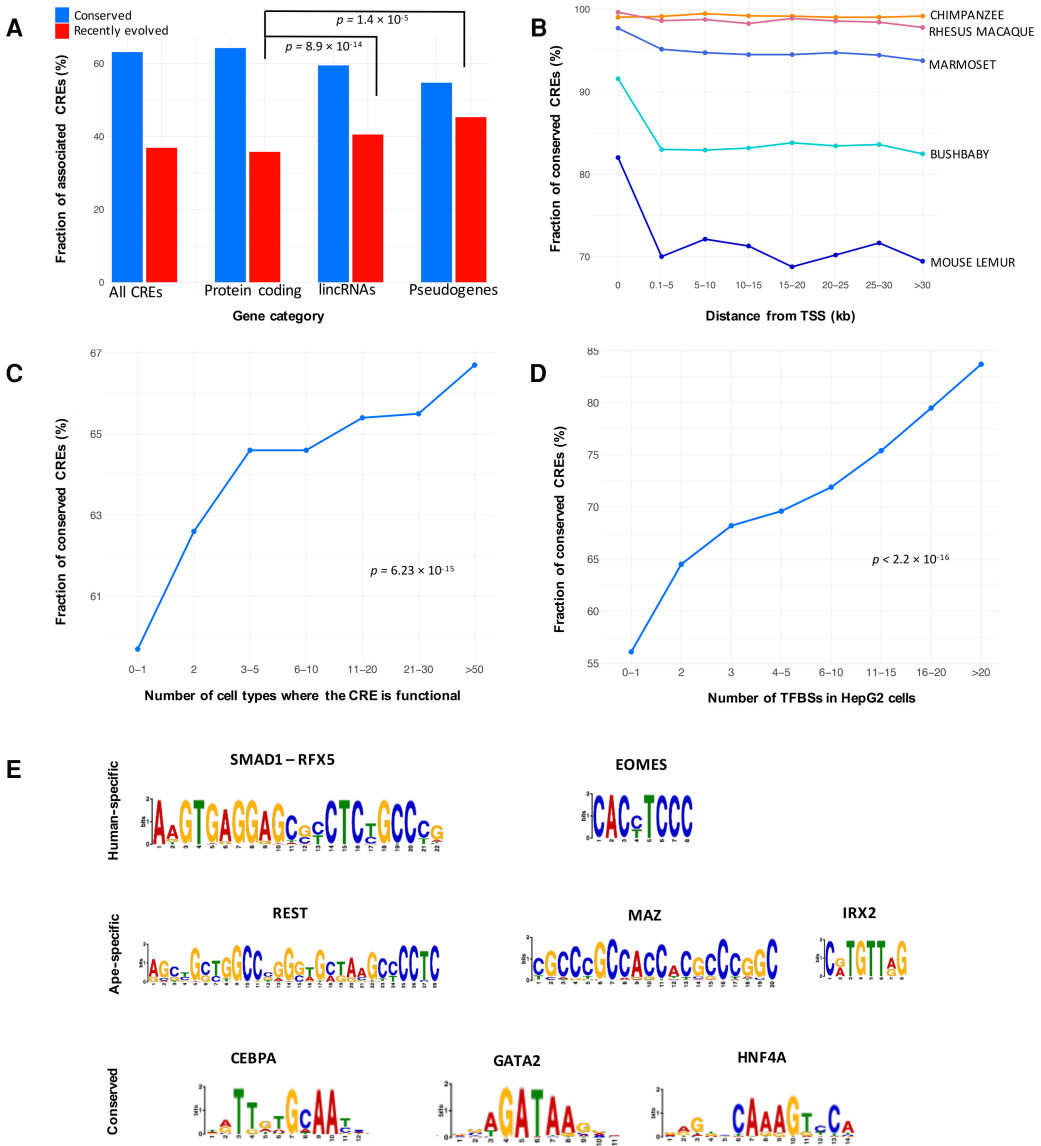
Genomic features associated with CRE conservation. (*A*) Fraction of conserved and recently evolved CREs associated with protein-coding genes, lincRNAs, and pseudogenes. (*B*) CRE conservation (*y*-axis) as a function of distance to the nearest gene start (quantiles on the *x*-axis). (*C*) CRE conservation (*y*-axis) as a function of cell-type specificity (quantiles on the *x*-axis) based on ENCODE data. (*D*) CRE conservation (*y*-axis) and number of ENCODE HepG2 TFBSs overlapping each CRE (quantiles on the *x*-axis). (*E*) Examples of enriched motifs in human-specific CREs, ape-specific CREs, and conserved CREs.

To understand the mechanisms responsible for CRE conservation and turnover, we identified genomic features associated with conserved regulatory activity. CREs associated with protein-coding genes were significantly more conserved than CREs associated with either pseudogenes (Fisher's exact test *P* = 1.4 × 10^−5^) or lincRNAs (Fisher's exact test *P* = 8.9 × 10^−14^) (Fig. 4A). For closely related species, regulatory activity was conserved, regardless of the distance to the nearest TSS (human × chimpanzee, logistic regression *P* = 0.30) (Fig. 4B). However, for more distantly related species pairs, the evolutionary conservation of the CRE activity was significantly lower in regions more distant from TSSs (human × rhesus macaque, logistic regression *P* < 2.2 × 10^−16^; human × marmoset, *P* < 2.2 × 10^−16^; human × bushbaby, *P* < 2.2 × 10^−16^, human × mouse lemur, *P* < 2.2 × 10^−16^) (Fig. 4B). Intronic enhancers were significantly more conserved than intergenic enhancers (64.2% and 55.8%, respectively; Fisher's exact test *P* < 2.2 × 10^−16^). These data demonstrate increased selective pressure to maintain regulatory activity in the vicinity of protein-coding genes.

Multiple genomic features indicative of broad regulatory element activity were significantly associated with regulatory conservation. Promoters and enhancers overlapping regions of chromatin accessibility in many cell types (The ENCODE Project Consortium 2012) were significantly more conserved than those that are functional in only a small number of cell types (logistic regression *P* = 6.2 × 10^−15^) (Fig. 4C). Similarly, conservation of CRE activity was significantly correlated to the number of transcription factor binding sites (TFBSs), as identified by ENCODE ChIP-seq in HepG2 cells (logistic regression *P* < 2.2 × 10^−16^) (Fig. 4D). A Gene Ontology analysis for genes associated with conserved CREs revealed enrichment for regulation of cellular, transcriptional, and developmental processes (Supplemental Table S4).

### Specific transcription factor motifs are associated with regulatory conservation and turnover

We used the MEME Suite (Bailey et al. 2009) to identify sequence motifs enriched in human-specific, ape-specific, and evolutionarily conserved liver CREs. Human-specific CREs are enriched with motifs for transcription factors (TFs) associated with immune response and hematopoietic maintenance (Fig. 4E; Supplemental File S2), such as RFX5, SMAD1, and EOMES. The rapid evolution of immune response genes and TFs is supported by many studies in vertebrates and in *Drosophila melanogaster*, demonstrating that while the central machinery of immune responses is strongly conserved, components of the extended molecular networks can evolve rapidly or diversify as a consequence of evolutionary competition between hosts and pathogens (Jansa et al. 2003; Vallender and Lahn 2004; Sackton et al. 2007; Obbard et al. 2009; Schadt 2009; Grueber et al. 2014; Lazzaro and Schneider 2014; Salazar-Jaramillo et al. 2014; Zak et al. 2014; Sironi et al. 2015; Wertheim 2015). Ape-specific CREs are enriched for binding sites of TFs involved in liver function but also in brain and neural system proliferation and development (Fig. 4E).

Evolutionarily conserved CREs are enriched with motifs for master regulators and homeobox genes that establish cell-type identity in liver cells (Fig. 4E; Supplemental File S2). Among these master regulators, HNF4A is essential for the differentiation of human hepatic progenitor cells (DeLaForest et al. 2011). Likewise, CEBPA is required for the liver cell specification and gene function, and the associated TFBSs are highly conserved across mammals (Ballester et al. 2014). Both CEBPA and HNF4A have conserved *cis*-regulatory activity and a large number of shared TF binding events across distant vertebrates (Schmidt et al. 2010). These results demonstrate that evolution shapes the regulatory landscape by preserving the regulatory activity in essential metabolic and developmental pathways, while permitting incessant renovation of specific networks that are under strong selective pressures.

### TE-derived CREs are pervasive in the primate genomes

**Figure 5.**
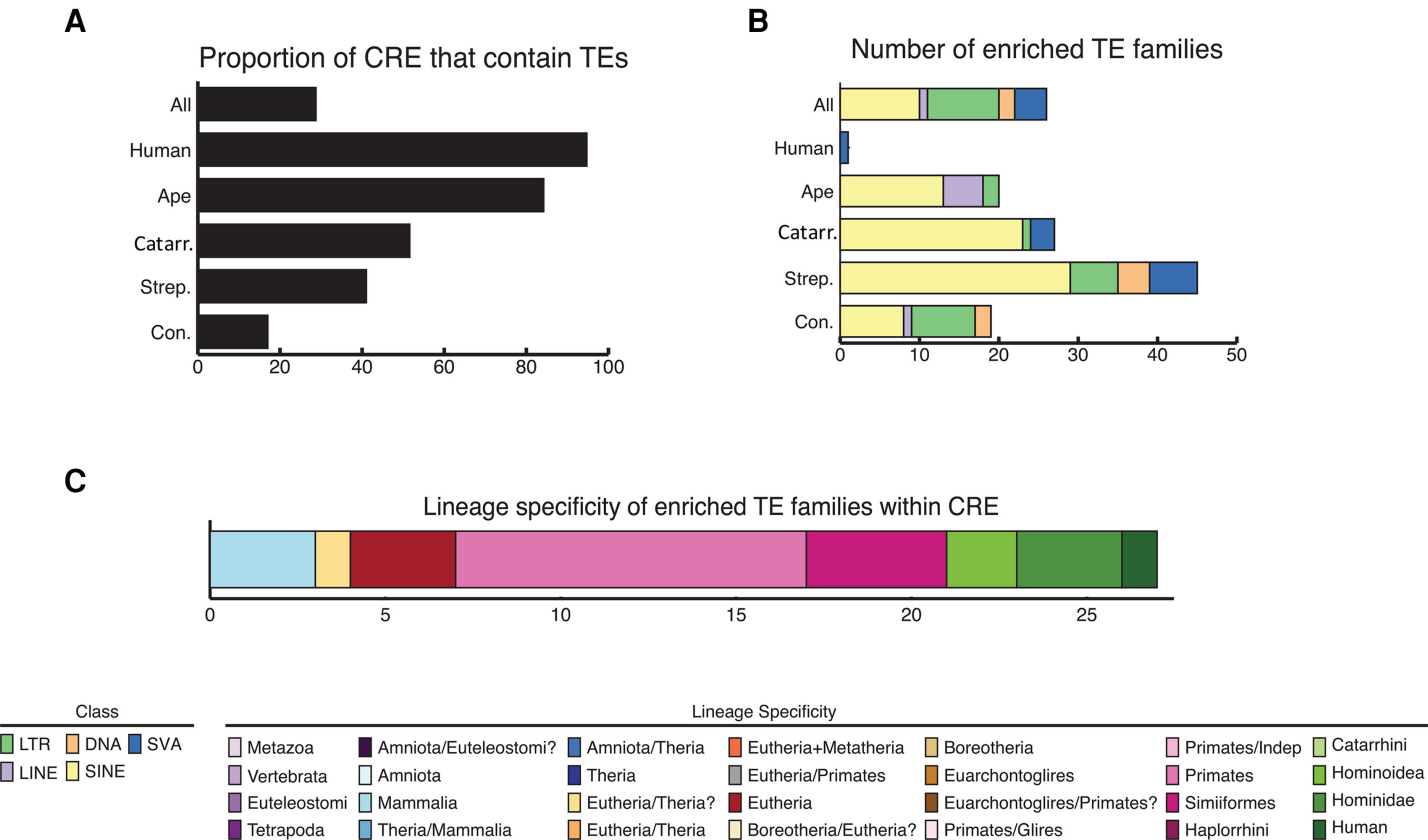
Newly evolved CREs are enriched in TEs. (*A*) Proportion of CREs that overlap TEs in the different primate lineages. (*B*) Number of enriched TE families within CREs in the different primate lineages. (*C*) Most enriched TE families in primates.

To quantify the contribution of TEs to the regulation of liver gene expression, we annotated each liver CRE based on overlap with RepeatMasker elements (Smit et al. 2013–2015): 9877 of the 39,710 six-way-alignable CREs, (24.9%) overlapped a TE for at least 20% of their length. A total of 24 TE families were significantly enriched in CREs (FDR < 1%) (Supplemental Table S5), nearly all of which were SINE-VNTR-*Alu*s (SVAs), and LTRs (mostly ERV1) (Fig. 5). As we filtered our ChIP-seq data for high-confidence alignments (see Methods), reads mapping to young TE families are likely underrepresented among our identified CREs (Supplemental Fig. S6). While only 0.01% of the human TEs overlap a human CRE, when restricting the analysis to the 39,710 six-way-alignable CREs, 73.5% of TE insertions are found within a differentially modified CRE (Fisher's exact test *P* < 2.2 × 10^−16^) (Supplemental Table S10).

The majority (75.0%) of enriched TE families were relatively young, and specific to humans (SVA-F), Hominidae (SVA-B, SVA-C, and SVA-D), Hominoidea (the LTR12 subfamily), Simiiformes (LTRs), or primates (*Alu* elements), whereas the remaining 25.0% were Eutherian-specific or older (Fig. 5; Supplemental Fig. S5; Supplemental Table S5). Given that young TE families are likely depleted from our alignments due to alignment limitations, we believe these estimates are likely conservative. TE enrichment analyses were based on the expected proportion of CRE that contain each kind of TE. Thus, SVAs, and also LTRs, were significantly more abundant than expected, regardless of the TE lineage specificity, despite being among the least common classes of repeats in the human genome (15.9% and 0.69% of the total TEs, respectively; Fisher's exact test *P* < 2.2 × 10^−16^ for both of the TE categories).

In at least one pairwise comparison, 57.3% of CREs harboring TEs were differentially active. Among these, SVAs (2.5%) were overrepresented (Fisher's exact test *P* < 2.2 × 10^−16^). We therefore investigated whether these recent TE insertions altered the expression patterns of nearby genes in primates. We focused on SVAs and LTR12-C,D,E TE categories present only in human and chimpanzee. Of genes associated with CREs overlapping either SVAs or LTR12-C,D,Es, 17.6% were differentially expressed between apes and the other tested primates. In contrast, genes associated with primate-specific TEs or not associated with any TE are significantly less likely to be differentially expressed between apes and non-ape primates (Fisher's exact test *P* < 0.002). Genes whose CREs recently acquired TE insertions did not exhibit greater within species expression variability than genes without TE insertions. This suggests that genes that acquire TE insertions are not simply more tolerant to variable expression (Supplemental File S4).

### The vast majority of recently evolved CREs are derived from TE insertions

Overall, 77.1% of ape-specific CREs and nearly all human-specific CREs overlap a TE (Fig. 5; Supplemental Fig. S5). In contrast, only 16.0% of evolutionarily conserved CREs contain an annotated TE. LTRs (in particular LTR-12C) and SVAs are the most common TEs overlapping newly evolved CREs (LTR = 40.1% of the recruited TEs in ape-specific CREs; SVA = 75.3% of the recruited TEs in the human-specific CREs) (Fig. 5). The regulatory function of nine CREs overlapping a TE were validated in our massively parallel reporter assay (MPRA) experiment, including five with ape-specific functions.

The contribution of LTRs to gene regulation has been proposed in previous studies (Wang et al. 2007; Cohen et al. 2009; Sundaram et al. 2014; Chuong et al. 2016; Janoušek et al. 2016). An example of an ape-specific CRE derived from LTR insertion is an enhancer at the gene *GRIN3A*. This gene is involved in physiological and pathological processes in the central nervous system and has been associated with several complex human diseases, including schizophrenia (Takata et al. 2013). *GRIN3A* is up-regulated in apes compared with other primates (log_2_ fold change = 2.00; FDR < 0.02) (Supplemental Fig. S3). Further, our differential histone modification analysis identified an ape-specific ChIP-seq peak overlapping a 1-kb-long ape-specific insertion (present also in orangutan and gorilla, but not in other primates; GRCh38 Chr 9: 101,723,127–101,724,197). This insertion, located 13 kb from the TSS of *GRIN3A*, is entirely derived from an LTR-12C. The insertion drove strong enhancer activity upon transfection into HepG2 cells (Wilcoxon rank-sum test *P* = 0.00017) (Supplemental Fig. S3), suggesting that the TE insertion results in a functional enhancer at the *GRIN3A* locus.

SVAs are a hominid-specific family of composite retrotransposons active in humans (Hancks and Kazazian 2010), with more than 3500 annotated copies. SVAs that overlap a liver CRE are significantly closer to the TSS of the associated gene than those that do not overlap a CRE (Wilcoxon rank-sum test *P* = 0.00117), suggesting that an SVA has a higher probability of becoming a CRE if it inserts near gene promoters (Supplemental Fig. S2).

Among the SVAs with significant histone modification, we identified an intronic CRE for the gene *JARID2.* This gene is an accessory component of Polycomb Repressive Complex-2 (PRC2), recruits PRC2 to chromatin, and is involved in liver, brain, neural tube development, and embryonic stem cell differentiation (Kaneko et al. 2014). Our differential histone state analysis identified a human-specific ChIP-seq peak overlapping a human-specific 1.9-kb-long insertion, entirely derived from an SVA-F human-specific retrotransposon. *JARID2* is significantly down-regulated in humans compared with all the other primates (log_2_ fold change = −3.33; FDR < 0.02) (Supplemental Fig. S4). SVAs-Fs overlapping a CRE exhibit significant enrichment for binding sites of known transcriptional repressors such as PAX5, FEV, and SREBF1 (Maurer et al. 2003; Fazio et al. 2008; Lecomte et al. 2010). Indeed, the *JARID2* SVA-F insertion leads to significantly decreased expression in HepG2 reporter assays (Wilcoxon rank-sum test *P* = 0.00275) (Supplemental Fig. S4). Furthermore, 78.3% of the genes associated to CREs with human-specific SVA insertions exhibit significantly decreased expression relative to their nonhuman orthologs (Fisher's exact test *P* < 1.6 × 10^−6^). This, along with additional validation assays presented below, supports the role of SVA insertions as transcriptional repressors.

### Broad regulatory activity of TE insertions in the primate liver

**Figure 6.**
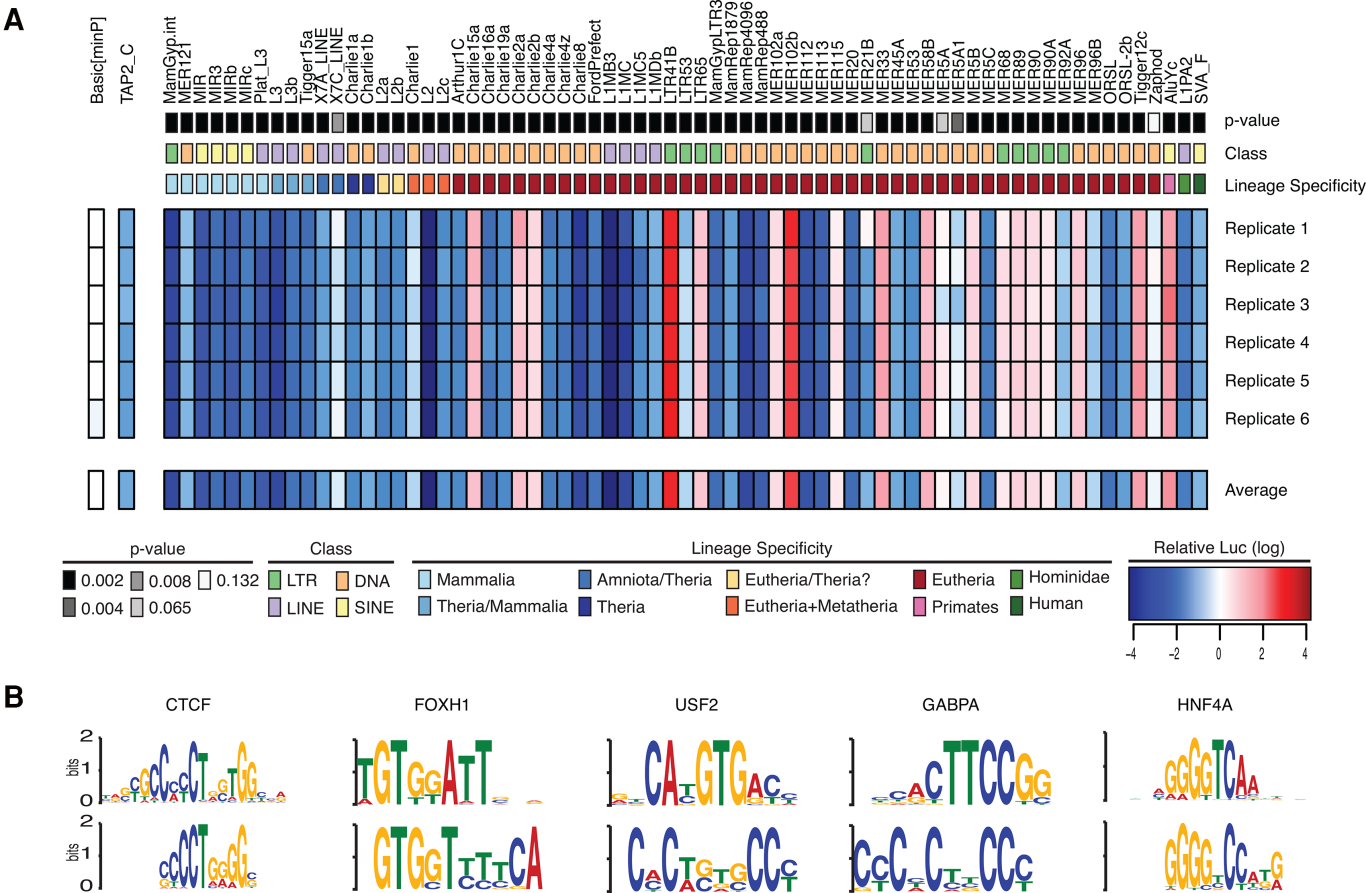
Regulatory ability of TE families found in poised and active regulatory elements in HepG2 cells. (*A*) The *P*-value (Wilcoxon rank-sum test), class, and lineage specificity for the 69 TE families tested in HepG2 cells for regulatory ability, the empty vector control (Basic[minP]), and the positive control (TAP2_C) are all shown *above* the six luciferase assay replicates conducted and the average regulatory ability found across the replicates. Red indicates luciferase expression higher than the empty vector control; blue indicates luciferase expression less than Basic[minP]. (*B*) Motifs enriched in the sequences of the 66 TE families that drive expression significantly different from background.

Our findings strongly suggest that the majority of novel CREs in primates are derived from TE insertions. To validate the predicted regulatory activity of recent TE insertions, we tested the *cis*-regulatory activity of 69 TE subfamilies, covering all major classes and families of primate TEs (Supplemental Table S6). TEs from these families overlap 3897 of our predicted CREs. We synthesized the mammalian consensus sequence for 69 different TE families, cloned them into a luciferase reporter vector with a minimal promoter, and transfected into HepG2 cells to perform luciferase reporter assays. Luciferase expression levels for 66 of the 69 (95.6%) tested TE families were significantly different from the negative control (Fig. 6A; Wilcoxon rank-sum test *P*-values in Supplemental Table S6). Strikingly, only 17 (25.7%) of these, mostly LTRs and DNA transposons, increased gene expression (Fig. 6A), whereas the remaining 49 (74.3%), mostly LINEs, repressed transcription. SVAs were confirmed as transcriptional repressors.

These findings demonstrate that LTRs, among the most enriched TEs in our peak set and the most common TE with a signature of regulatory function in apes, frequently result in increased regulatory activity. LTRs are known to have strong regulatory elements (Chuong et al. 2016). While only 2% of all human LTRs are marked by active histone modification, 25.5% of genes with an LTR insertion in an associated CRE are differentially expressed, suggesting that the effects of LTR insertions on local gene expression are strongly context specific.

The consensus sequences for the 66 TEs that significantly affected reporter expression were analyzed with MEME to identify enriched motifs. Motifs for known master regulators of liver cell identity, including FOX, USF2, GABP, and HNF4A (Wallerman et al. 2009), were significantly enriched within the sequences of the 66 TE families with significant regulatory activity (Fig. 6B). In summary, most TE families are capable of functioning as CREs in the primate liver, either as enhancers or repressors, further supporting our findings on the pervasive involvement of TEs in the primate gene regulation.

## Discussion

Less than 1% of tested CREs resulted as differentially active between humans and chimpanzees. This suggests that even modest changes in gene regulation produce significant differences, and confirms that *cis*-regulatory evolution plays a central role in primate diversification (Davidson 2001, 2006; Wray 2007; Ho et al. 2009; Tsankov et al. 2010; Martin et al. 2012; Coolon et al. 2014; Martin and Reed 2014; Guo et al. 2015; Lynch et al. 2015; Villar et al. 2015; Adachi et al. 2016; Landeen et al. 2016; Lesch et al. 2016; Zhang and Reed 2016).

Our approach for the comparison of CREs across species, based on the analysis of differential histone modification state in orthologous regions, demonstrated that *cis*-regulatory divergence across species may be overestimated when assessed based on binary peak overlap. The increased density of primate sampling in our data set, compared with previous studies, allowed us to address the timing of primate regulatory divergence and improved the interpretation of when, and how, different TE families have been recruited for regulatory function. Generating transcriptome data from the same specimens that were epigenetically profiled allowed us to directly measure the effects of conserved and recently evolved CREs on the expression levels of the primary liver tissue.

With a combination of three different reporter assay techniques, we have tested and validated the regulatory activity of thousands of predicted CREs and TE insertions. Although previous studies have suggested the recruitment of TEs as functional elements (Huda et al. 2010; Lynch et al. 2011, 2015; Jacques et al. 2013; Chuong et al. 2016), we demonstrated, and functionally validated, the extent of this phenomenon in primates, demonstrating that LTRs and SVAs have played an important role in rewiring ape gene regulation. In contrast, only a minor fraction of evolutionarily conserved CREs overlap an annotated TE. Together, our data suggest that the core regulatory network that establishes liver-cell-type identity in primates is conserved, whereas rapid evolution occurs on the periphery of the network, where TEs have the most impact on gene regulatory evolution.

## Methods

### Tissue sampling

We obtained liver tissue samples for three or four individuals belonging to each of the studied species (Supplemental Table S1) from Texas Biomedical Research Institute and from Duke University Lemur Center. Samples were collected and flash-frozen immediately.

### RNA-seq sample processing

We processed samples from all species in random batches of four to minimize batch effects. We used 4 µg of total RNA to produce barcoded RNA sequencing libraries using the Illumina TruSeq stranded mRNA kit (Supplemental File S4). Libraries were pooled in two different pools based on barcode compatibility, and each pool was sequenced on two Illumina HiSeq 2500 lanes, producing on an average of 42.1 million single-end 100-bp reads per sample.

### ChIP-seq sample processing

We processed samples in six randomly assigned groups in order to minimize batch effects (Supplemental File S4). We used 5–15 ng of input and immunoprecipitated DNA to generate sequencing libraries using the NEBNext Ultra ChIP-seq library kit. Libraries were multiplexed, pooled, and sequenced on a total of 16 Illumina HiSeq 2500 lanes, producing on an average of 40.6 million SE 100-bp reads per sample.

### Sequence QC: ChIP-seq and RNA-seq

We assessed standard QC measures on FASTQ files using FASTQC v0.11.3 (http://www.bioinformatics.babraham.ac.uk/projects/fastqc). We trimmed sequencing adapters and low-quality base calls using Trim Galore! v0.4.1 (http://www.bioinformatics.babraham.ac.uk/projects/trim_galore/).

### RNA-seq alignment and gene expression quantification

We aligned all sequences that passed QC to the reference genomes from the Ensembl database release 87 (bushbaby: otoGar3; chimp: CHIMP2.1.4; humans: GRCh38; rhesus macaque: Mmul1; marmoset: C_jacchus3.2.1; mouse lemur: Mmur1) using STAR v2.5, in 2-pass mode (Dobin et al. 2013; Supplemental File S4). We used featureCounts (Liao et al. 2014) to count reads mapping to each gene, according to Ensembl annotations for the six studied species.

### Differential gene expression analysis

We analyzed differential gene expression levels using read counts, normalized by feature length with DESeq2 (Love et al. 2014), with the following model: *design* = ∼*condition*, where condition indicates the species or the group of species (e.g., apes).

We used a set of 10,243 genes annotated as orthologs in the six species according to Ensembl (BioMart v. 0.9) (Supplemental Table S3; Smedley et al. 2015) and used 5% false-discovery rate (FDR; Benjamini–Hochberg) (Benjamini and Hochberg 1995) as our multiple-testing-corrected significance threshold. The overall analysis included five comparisons: (1) human versus each of the other five species, (2) human-specific differential expression (human vs. other five primates grouped together), (3) ape-specific differential expression (human + chimpanzee vs. other four primates), (4) Catarrhini-specific differential expression (human + chimpanzee + rhesus macaque vs. other primates), and (5) comparison between Haplorrhini (human, chimpanzee, rhesus macaque, and marmoset) and Strepsirrhini (mouse lemur and bushbaby).

### ChIP-seq QC and alignment

We aligned the sequences that passed QC to the reference genomes from the Ensembl database (bushbaby: otoGar3; chimp: CHIMP2.1.4; humans: GRCh38; rhesus macaque: Mmul8.0.1; marmoset: C_jacchus3.2.1; mouse lemur: Mmur2), using Burrows–Wheeler alignment tool (BWA), with the MEM algorithm (Li 2013). Aligned reads were filtered based on mapping quality (MAPQ > 10) to restrict our analysis to higher quality and likely uniquely mapped reads, and PCR duplicates were removed.

### ChIP-seq peak calling and QC

We called peaks for each individual using MACS2, at 5% FDR, with parameters recommended for histone modifications (see https:// github.com/taoliu/MACS/wiki/Call-differential-binding-events): --no model --ext size 147 -B. We performed QC on peaks called for each specimen using metrics recommended by ENCODE (Landt et al. 2012; Supplemental File S4). Samples that did not pass the three main QC metrics (FRiP, NSC, RSC) were excluded for any downstream analysis. We called human consensus peaks for H3K27ac and H3K4me1 using MACS2 and the above-described parameters. All human samples passing QC were considered as replicates for the consensus peak calling. The human consensus H3K27ac and H3K4me1 peaks were used to perform all human-centric downstream analyses.

### Parallelized reporter assay

We obtained a list of 334 putative 1-kb-long CREs overlapping liver eQTLs (Brown et al. 2013). Two hundred seventy-six out of these 334 CREs overlapped one of our human peaks (96 enhancers and 180 promoters) (Supplemental Table S6). Within each of the loci defined by the 276 liver eQTLs, we predicted a 1-kb CRE, and tested their functionality as described in Supplemental File S4.

### Detection of orthologous regions for human peaks in each primate

We mapped orthologous sequences using all identified human consensus ChIP-seq peak regions in both the H3K27ac and H3K4me1 experiments using the 40 Eutherian mammals Ensembl MSA. The detailed pipeline is illustrated in Supplemental File S4.

### Differential histone modification analysis

By use of the above-described procedure, for both H3K27ac and H3K4me1, we produced a single matrix including the human peaks having an ortholog in each of the studied species, as well as the associated read count for each histone mark plus the input in all of the six species. Read counts were used for differential ChIP-seq analysis with DESeq2, performing an interaction analysis between the histone marks read counts and their associated input values, using the Wald statistic: *design* = ∼*assay* + *condition* + *assay:condition*, where the assay indicates either IP data or input data, and condition indicates the species or the group of species.

Differential histone mark analysis included the same species × species and group × group comparisons described for RNA-seq (FDR < 10%). Further, different FDRs (up to 50%) were tested to assess the robustness of our approach. We initially analyzed differential histone modifications for the two marks independently. Then, overlapping CREs were merged.

### Sequence conservation

We estimated per-nucleotide pairwise divergence for all five species in comparison to humans using the MSA aligned sequences of orthologous regions for consensus peaks ±500 bp. All gaps in human were excluded from analysis. Regions not included in the set of six-way orthologous CREs were pruned. Finally, we removed outliers—with respect to the distribution of the genetic distances in the given pairwise comparison—using the R package *outliers* (Komsta 2006).

### TE enrichment

TE enrichment analysis was performed using the *TEAnalysis* pipeline with TE-analysis_Shuffle_bed v. 2.0, setting 1000 replicates (Kapusta et al. 2013; https://github.com/4ureliek/TEanalysis).

### Luciferase reporter assay validation of *GRIN3A* and *JARID2*

We compared activity of two predicted functional CREs with the empty pGL4.23 vector as a negative control, as described in the Supplemental File S4.

### Validation of the gene regulatory functionality of TE families

TE constructs were built by synthesizing (GenScript) the Dfam (Hubley et al. 2016) consensus sequence for 69 TE subfamilies, representing all of the major TE classes and families. Each element was cloned into pGL3 Basic vector (Promega) with an added minimal promoter (pGL3 Basic[minP]). Luciferase assays were performed as described in the Supplemental File S4.

### Statistical and genomic analyses

All statistical analyses were performed using R v3.3.1 (R Core Team 2016). Figures were made with the package ggplot2 (Wickham 2009). BEDTools v2.25.0 (Quinlan and Hall 2010) was used for genomic analyses. Scripts and pipelines are available online (https://github.com/ypar/cre_evo_primates.git) and in the Supplemental File S5.

## Data access

All raw sequence data from this study have been submitted to the NCBI BioProject database (BioProject; https://www.ncbi.nlm.nih.gov/bioproject/) under accession numbers PRJNA349047 (RNA-seq) and PRJNA349046 (ChIP-seq).

## Supplementary Material

Supplemental Material
