# Supplementary material for: Transposable elements are the primary source of novelty in primate gene regulation
Source: Genome Res. 2017 Oct;27(10):1623–33. doi: 10.1101/gr.218149.116 (PMC5630026; doi:10.1101/gr.218149.116)
Supplement: Supplemental Material [file supp_27_10_1623__index.html]

Transposable elements are the primary source of novelty in primate gene regulation — Supplemental Material 

# Transposable elements are the primary source of novelty in primate gene regulation

## Supplemental Material

- Supplemental\_figures\_with\_captions.pdf
- Supplemental\_FILE\_S1\_peak\_calling\_BED\_files.zip
- Supplemental\_FILE\_S2\_MOTIF\_ANALYSES\_OUTPUTS.zip
- Supplemental\_FILE\_S3.zip
- Supplemental\_FILE\_S4.pdf
- Supplemental\_FILE\_S5.pdf
- Supplemental\_TABLE\_S1.xlsx
- Supplemental\_TABLE\_S2.csv
- Supplemental\_TABLE\_S3.csv
- Supplemental\_Table\_S4.xls
- Supplemental\_TABLE\_S5.txt
- Supplemental\_TABLE\_S6.xlsx
- Supplemental\_TABLE\_S7.xlsx
- Supplemental\_Table\_S8.xlsx
- Supplemental\_Table\_S9.xlsx
- Supplemental\_Table\_S10.xlsx
- Supplemental\_Table\_S11.csv
