# Supplementary material for: Transposable elements are the primary source of novelty in primate gene regulation
Source: Genome Res. 2017 Oct;27(10):1623–33. doi: 10.1101/gr.218149.116 (PMC5630026; doi:10.1101/gr.218149.116)
Supplement: Supplemental Material [file supp_gr.218149.116_Supplemental_figures_with_captions.pdf]

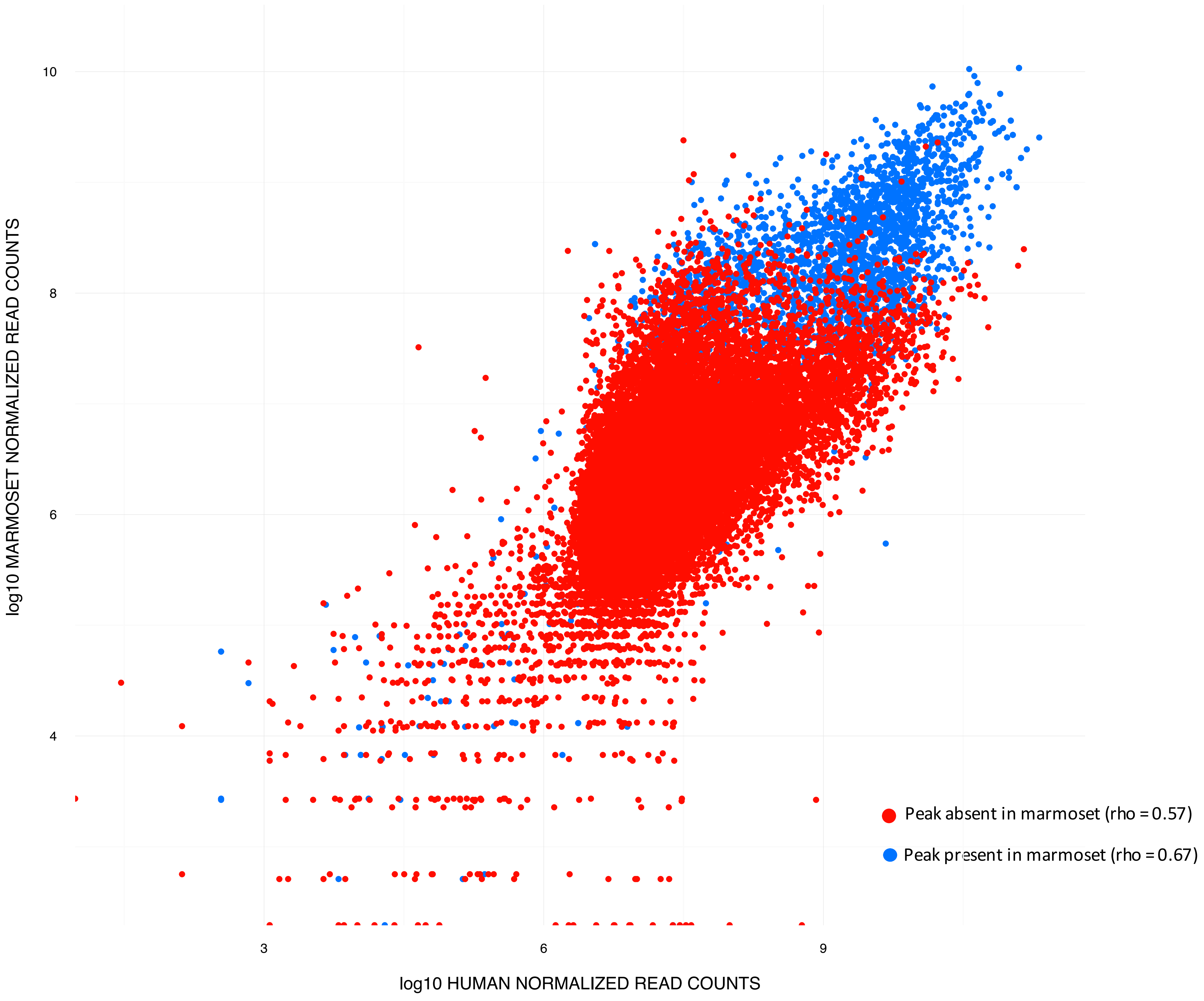

Figure S1: Comparison of the normalized ChIP-seq read counts between human and marmoset. Human consensus peaks with orthologs in all the six species were split into two groups for this analysis: 1) regions with overlapping peaks present in both marmoset and human, and 2) regions with a peak present only in human. While human and marmoset normalized read counts were more highly correlated with each other in group 1 (Spearman's ρ = 0.67; $p < 2.2\times10^{-16}$), we found a nearly as strong correlation in group 2 (Spearman's ρ = 0.57; $p < 2.2\times10^{-16}$). These findings are consistent with the results of our differential histone modification state analyses, which demonstrated that only a small fraction of the 39,710 orthologous CREs (5.15%, FDR < 10%) are differentially modified, despite the fact that we had a much smaller total number of peak calls in the marmoset samples.

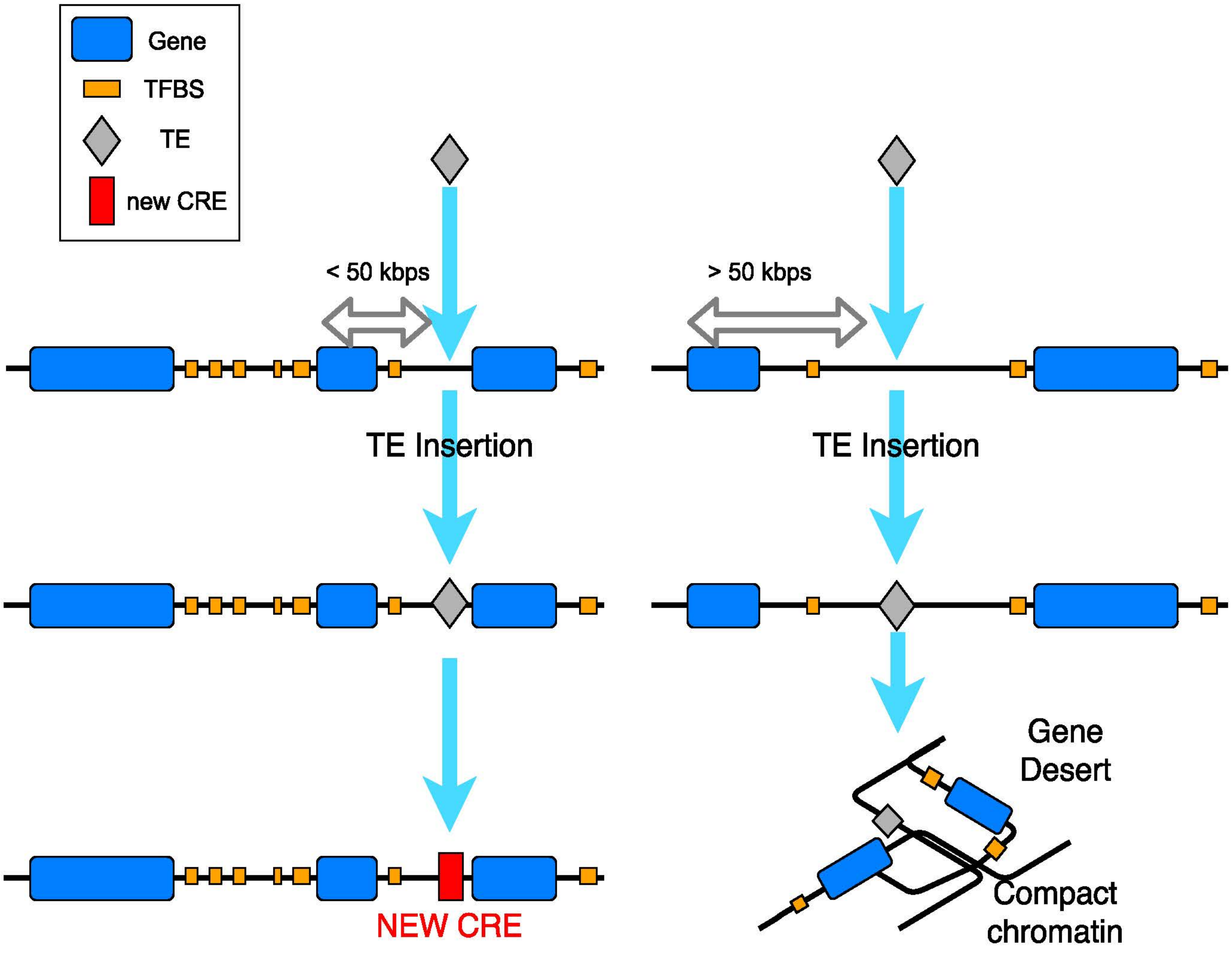

Figure S2: **Model for recruitment of TEs into human functional CREs**. A given TE has a higher probability of being recruited as a functional cis-regulatory element if it is found within 60 kb from a protein coding gene.

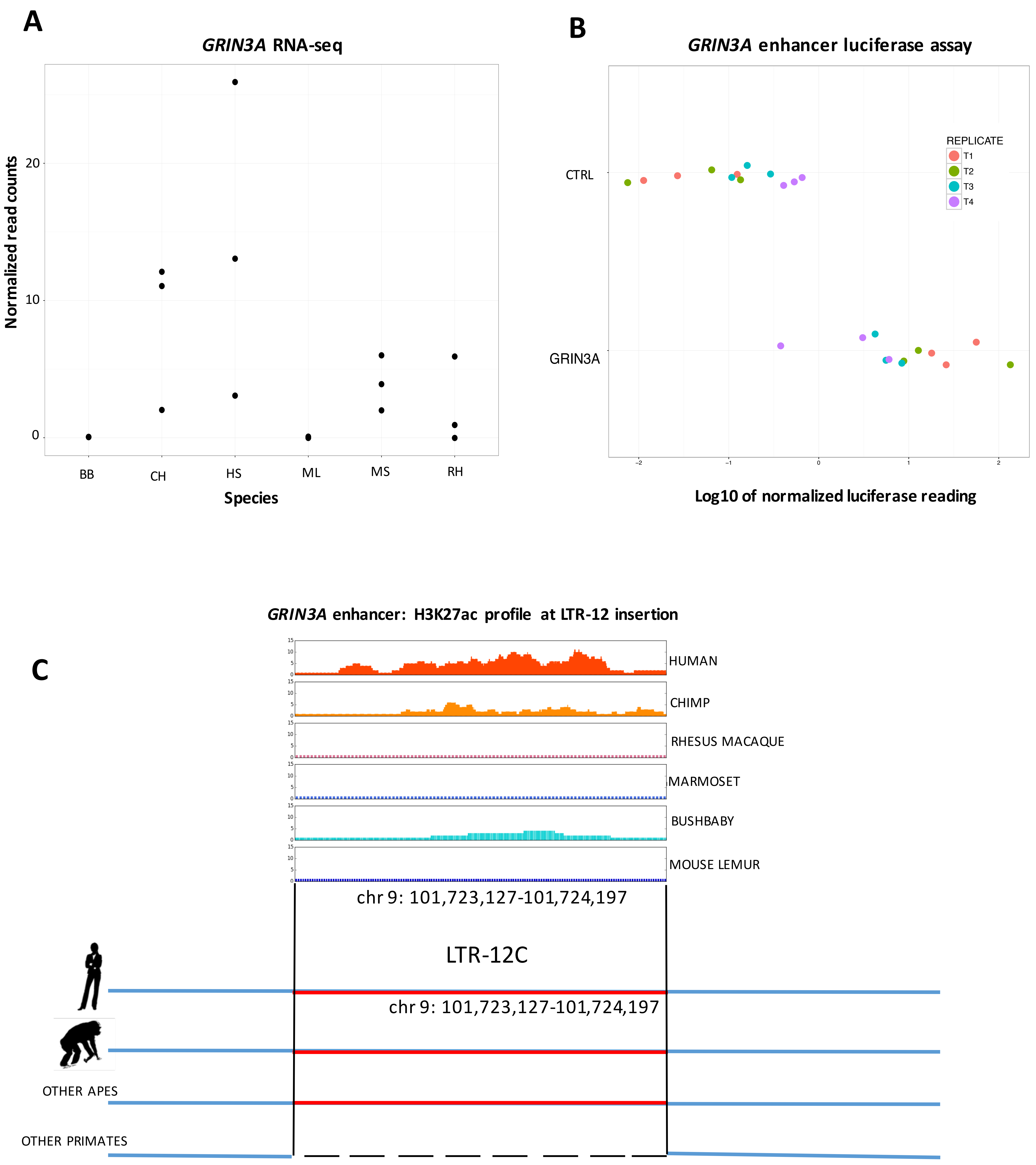

Figure S3: **Functional analysis on *GRIN3A locus*.** (A) RNA-seq data for *GRIN3A* (X= Species replicates; Y= RNA-seq read depth normalized by sequencing depth and gene length). (B) Luciferase assays reporter activity for the CREs associated to *GRIN3A* (X= $\log_{10}$[luciferase reading]; Y=sample processed [CTRL=vector with no insert; *GRIN3A* = vector with LTR insertion]).

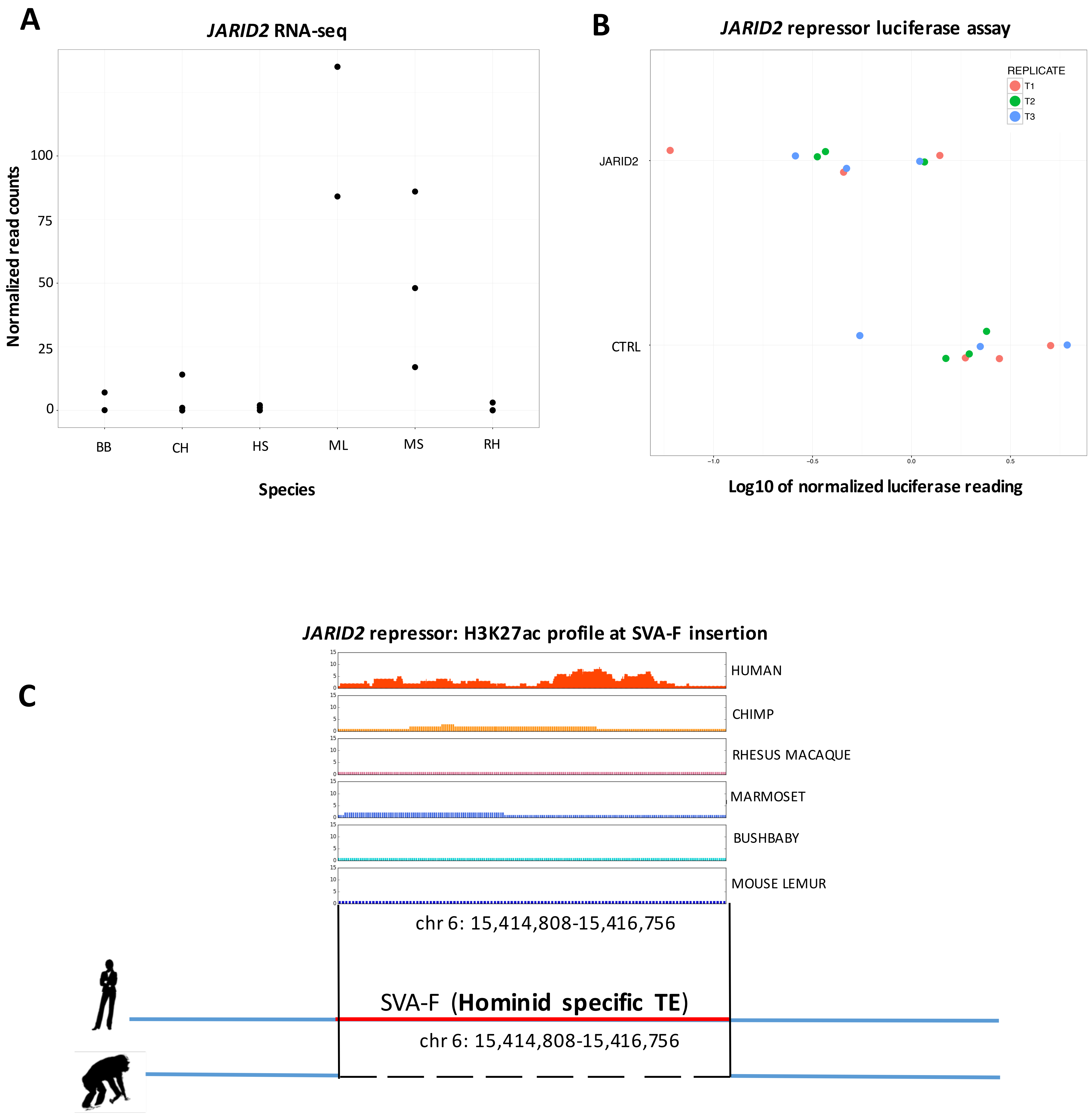

Figure S4: **Functional analysis on *JARID2 locus*.** (A) RNA-seq data for *JARID2* (X= Species replicates; Y= RNA-seq read depth normalized by sequencing depth and gene length). (B) Luciferase assays reporter activity for the CREs associated to *JARID2* (X= log_{10}[luciferase reading]; Y=sample processed [CTRL=vector with no insert; *JARID2* = vector with SVA insertion]).

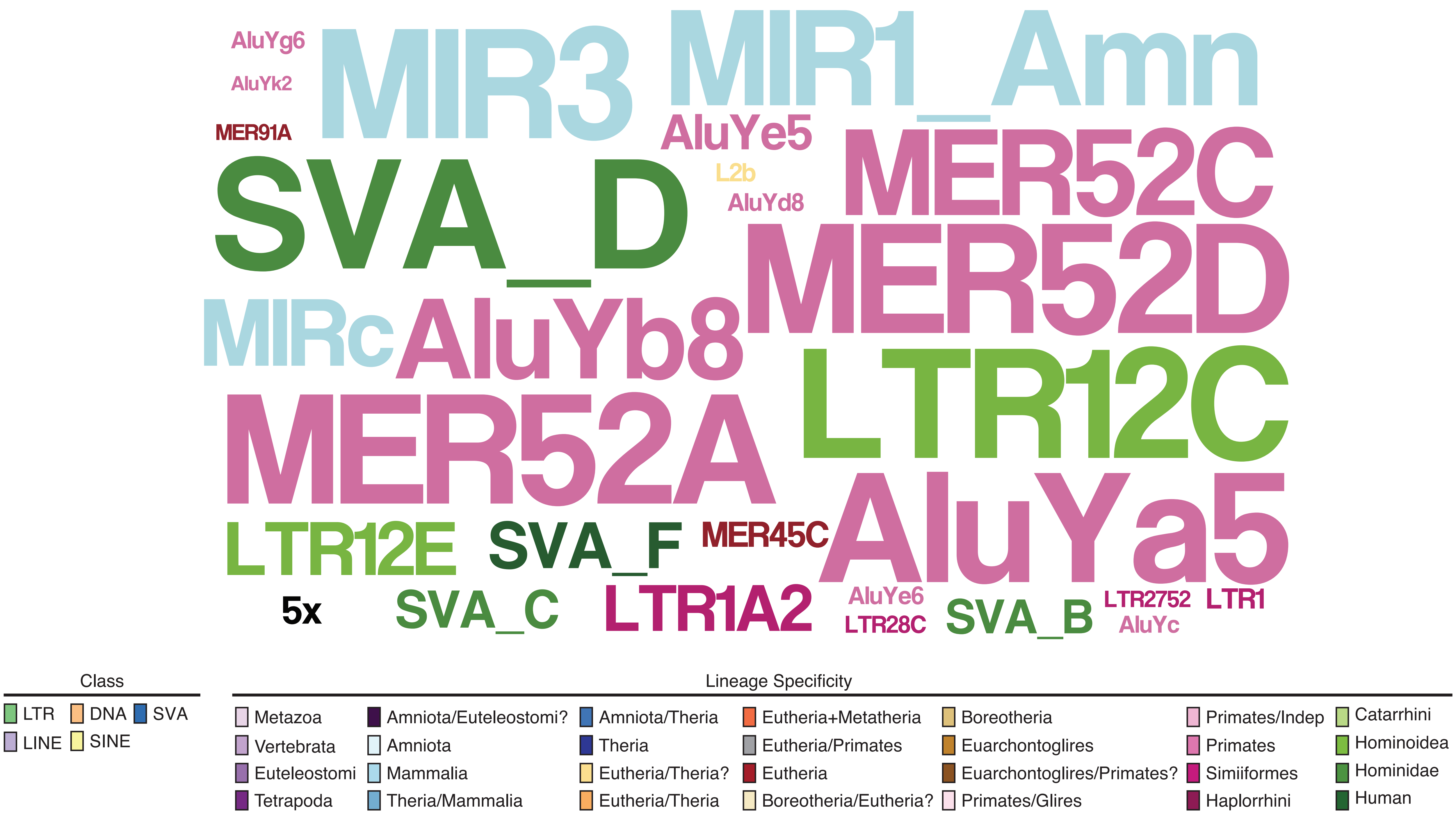

Figure S5: **Lineage specificity of enriched TEs.** Word-cloud representing the lineage specificity of the enriched TE families.

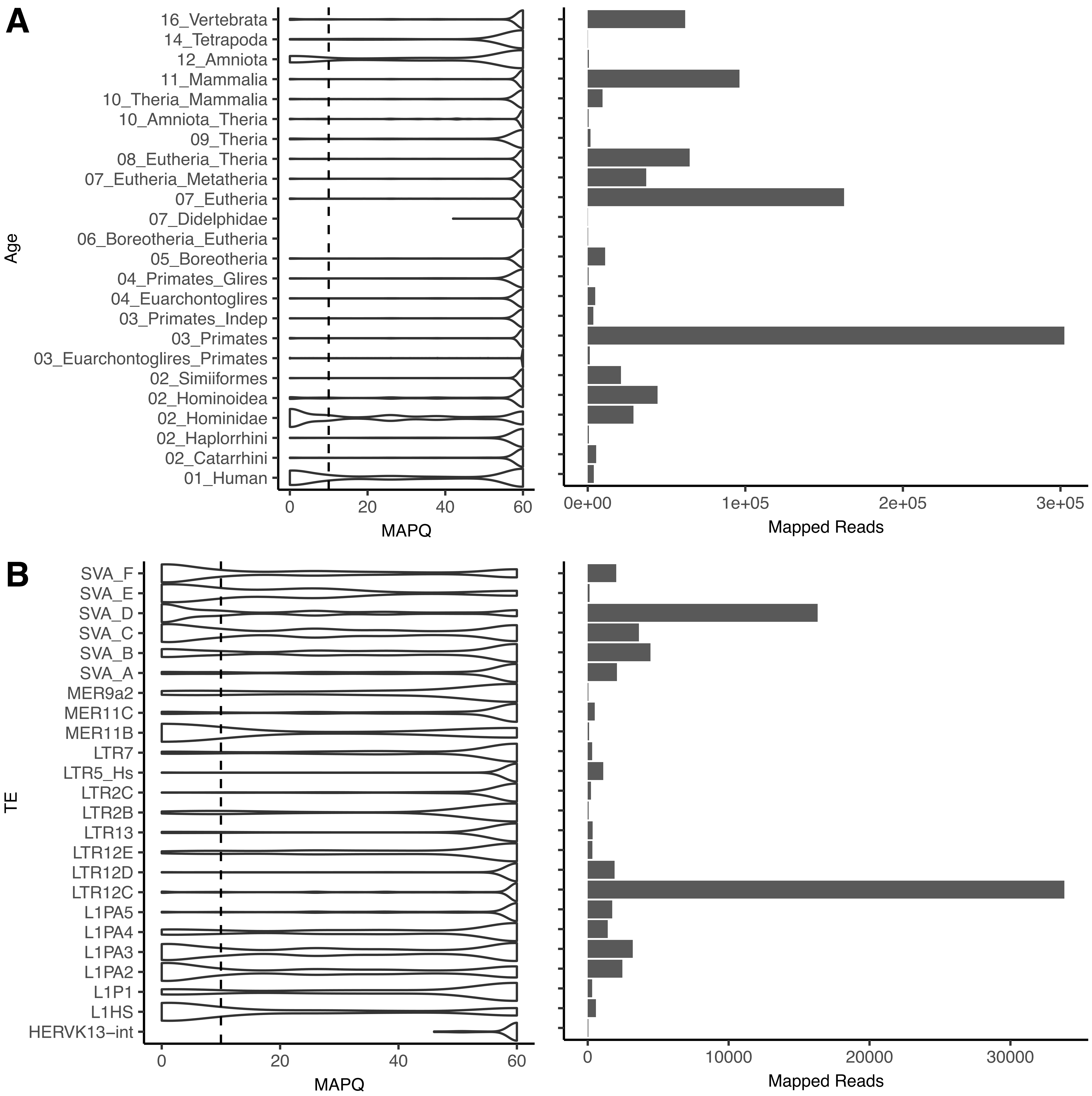

Figure S6: (A) Violin plot showing MAPQ for different TE taxonomic categories. (B) Violin plot showing MAPQ for human and hominid specific TEs.

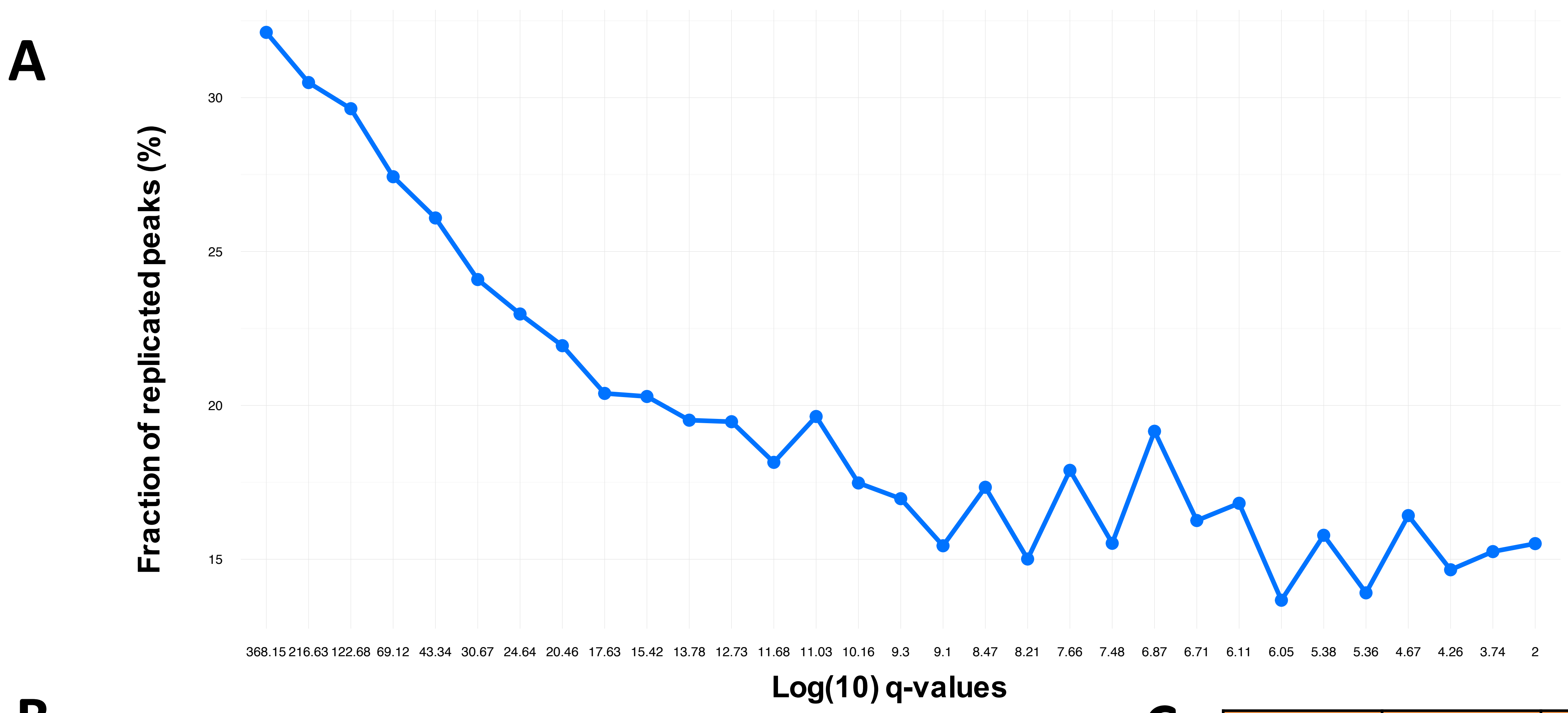

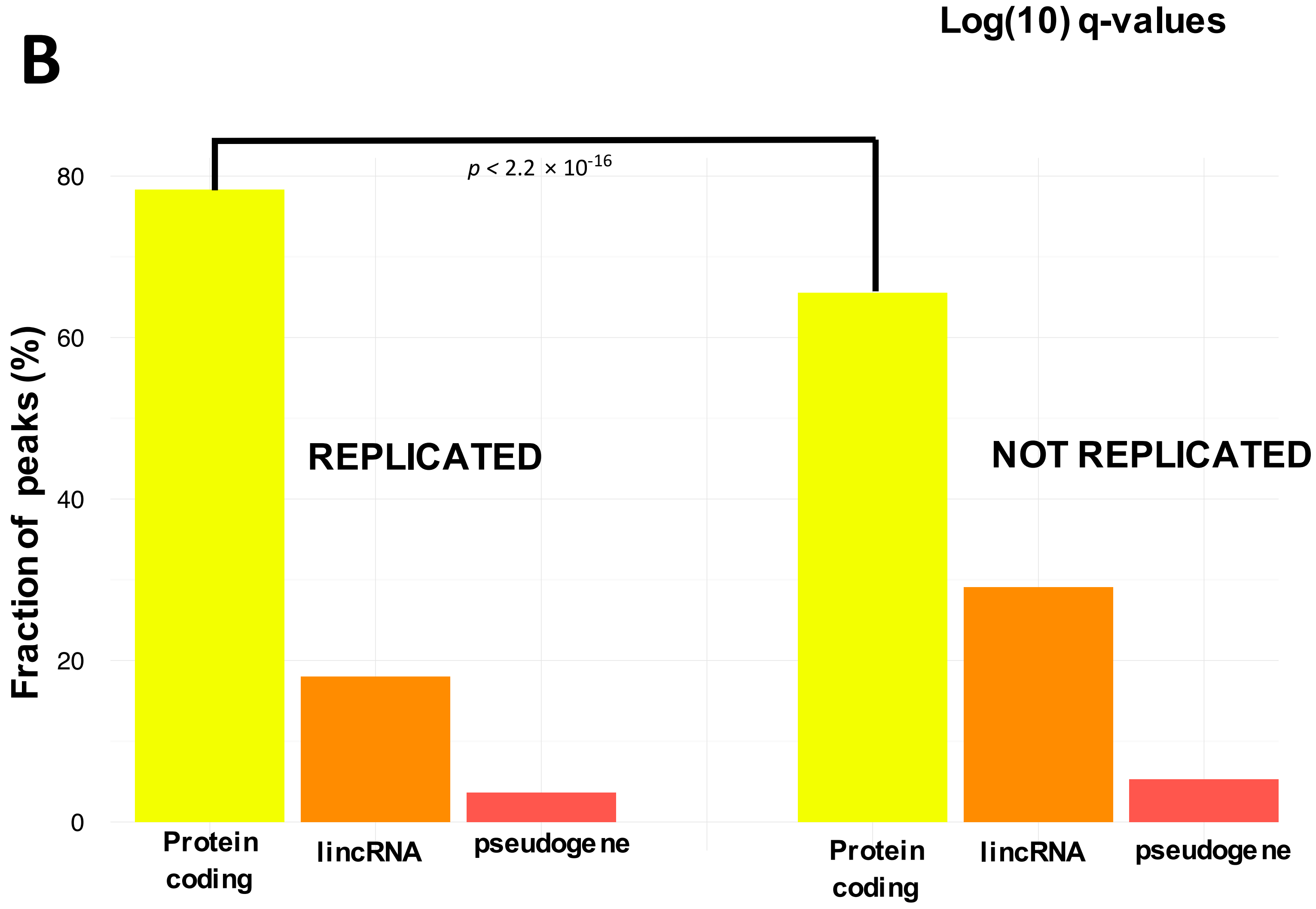

C

	REPLICATED	UNIQUE TO THIS STUDY	UNIQUE TO VILLAR
Distance from TSS (Kb)	7.6	21.8	30.3
Number of ENCODE cell types	11	4	6
Number of ENCODE HepG2 TFBS	3	2	2

Figure S7: (A) Correlation between fraction of peaks replicated in both this study and Villar et al 2015 study, and the q-value from peak calling. (B) Typology of genes associated to replicated and not replicated peaks. (C) For each group (REPLICATED PEAKS, UNIQUE TO THIS STUDY AND UNIQUE TO VILLAR ET AL), we have quantified the median (1) distance to the nearest gene, (2) number of cell types with an overlapping DHS site, and (3) number of TFs bound in HepG2 cells.
