# Supplementary material for: Transposable elements are the primary source of novelty in primate gene regulation
Source: Genome Res. 2017 Oct;27(10):1623–33. doi: 10.1101/gr.218149.116 (PMC5630026; doi:10.1101/gr.218149.116)
Supplement: Supplemental Material [file supp_gr.218149.116_Supplemental_FILE_S2_MOTIF_ANALYSES_OUTPUTS.zip › FILE_S2/conserved_hocomoco10_ame.html]

AME results


The name of the database (file name) that contains the motif.

[
close ]

A name for the motif that is unique in the motif database file.

[
close ]

An alternate name of the motif that may be provided in the motif database file.

[
close ]

For statistical test methods, splitting the sorted input sequences into two partitions at this rank gives the optimal *p*-value.
Sequences at and above this rank were treated as "positives", and sequences with larger rank
were treated as "negatives" when applying the statistical test that gives the optimal *p*-value.
For `linreg`), the optimal split is the one that yields
the minimum mean squared error.

[
close ]

The (optimal) enrichment *p*-value of the motif according to the statistical test.

[
close ]

The adjusted (optimal) enrichment *p*-value of the motif according to the statistical test.
This is the best *p*-value for the motif, adjusted for multiple tests using a Bonferroni
correction. The number of multiple tests is given at the start of this section. If the
*p*-value is *p* and the number of multiple tests is *n*, then the adjusted
*p*-value is 1 - (1-*p*)\*\**n*.

[
close ]

The value of Spearman's rank correlation coefficient (ρ). It varies between -1 and +1.
Low values of ρ indicate high correlation between the ranks of sequences
sorted by motif scores (decreasing) or FASTA scores (increasing). So,
assuming small FASTA scores are "good", a low value of ρ indicates that
a motif is enriched in the "good" sequences.

[
close ]

The value of the mean squared error of the linear regression of X vs Y.

[
close ]

The slope of the regression line: Y = mX + b.

[
close ]

The Y-intercept of the regression line: Y = mX + b.

[
close ]

# AME

## Analysis of Motif Enrichment

For further information on how to interpret these results or to get a
copy of the MEME software please access
http://meme-suite.org.

If you use AME in your research, please cite the following paper:  

Robert McLeay and Timothy L. Bailey,
"Motif Enrichment Analysis: A unified framework and method evaluation",
*BMC Bioinformatics*, **11**:165, 2010, doi:10.1186/1471-2105-11-165.
[full text]

Enriched Motifs
  |  
Input Files
  |  
Program information


# Javascript is required to view these results!


## Enriched Motifs

| Logo | Database | ID | Name | Split | *p*-value | Adjusted *p*-value | Spearman's Rank CC (ρ) | Mean squared error | m | b |
| --- | --- | --- | --- | --- | --- | --- | --- | --- | --- | --- |

## Input Files

#### Sequences

| Primary Sequences | Number ||

| --- | --- |

#### Motifs

| Database | Source | Motif Count |
| --- | --- | --- |

##### AME version

(Release date: )  
Copyright © Robert McLeay
r.mcleay@imb.uq.edu.au &
Timothy Bailey
t.bailey@imb.uq.edu.au, 2009.

##### Command line summary
