# Supplementary material for: Transposable elements are the primary source of novelty in primate gene regulation
Source: Genome Res. 2017 Oct;27(10):1623–33. doi: 10.1101/gr.218149.116 (PMC5630026; doi:10.1101/gr.218149.116)
Supplement: Supplemental Material [file supp_gr.218149.116_Supplemental_FILE_S4.pdf]

File S4: Supplemental Text (Supplemental Results and Methods)

Supplemental results

Binary peak overlap leads to an inflated estimate of CRE divergence across species.

First, human consensus peaks with orthologous in all the six species were split into two groups for this analysis: 1) regions with overlapping peaks present in both marmoset and human, and 2) regions with a peak present only in human. While human and marmoset normalized read counts were more highly correlated with each other in group 1 (Spearman's $\rho = 0.67$; $p < 2.2\times10^{-16}$), we found a nearly as strong correlation in group 2 (Spearman's $\rho = 0.57$; $p < 2.2\times10^{-16}$). These findings are consistent with the results of our differential histone modification state analyses, which demonstrated that only a small fraction of the 39,710 orthologous CREs (5.15%, FDR < 10%) are differentially modified, despite the fact that we had a much smaller total number of peak calls in the marmoset samples.

Next, we compared our H3K27ac data to a recent study focused on liver CREs in mammals (Villar et al., 2015). Based on binary peak overlap, only 53% of human peaks produced by Villar and collaborators (2015) overlap a human peak from our study, despite both peak sets having a comparable level of overlap with ENCODE HepG2 H3K27ac peaks (59% of ENCODE peaks overlaps a peak in our peak set, 63% in Villar et al.). Similarly, we compared our rhesus macaque and marmoset H3K27ac peaks with those produced by Villar et al. (2015), and found that 77% of macaque and 65% of marmoset peaks overlap between the two studies. To examine the incomplete overlap between the two human datasets, we have quantified differential histone modification using the same DESeq2 pipeline we used for inter species comparisons. We found that 89.1% (FDR 5%; 94.2% at FDR1%) of our human peaks do not show any differential histone marking in the two datasets.

CRE conservation across mammals

We assessed the extent to which primate-conserved CREs identified in this study are also evolutionarily conserved across a broader range of mammals. In particular, we compared our conserved H3K27ac CREs with the H3K27ac profile of the opossum, the species with the earliest divergence from humans (>180 million years) in the Villar et al. (2015) dataset. 2,854 primate-conserved promoters and 9,456 primate-conserved enhancers have orthologous regions in the opossum genome. Among these, 71.3% of the promoters and 19.1% of the enhancers had significant H3K27ac enrichment in both species, supporting that most of the primate conserved promoters show conserved activity in all of the mammalian clade, whereas only a fraction of the primate conserved enhancers are also conserved across mammals. Further, the two studies come to consistent estimates of the fraction of differentially active CREs per million years: 0.06–0.12% in primates and 0.07% in mammals.

Effect of TEs on gene expression

We tested whether the effect of TE insertions on gene expression might be simply due to the fact that genes with more variable expression are more tolerant of TE insertions. We thus assessed within species variability in expression for all non-human species, and performed poisson regression on each gene with species as a covariate. We have then performed a Wilcoxon rank sum test on the summed square of these residuals, for each gene, stratified by the presence or absence of a human TE insertion overlapping a CRE. For example, when stratified by the presence of a human specific transposon insertion, we saw no significant difference in within species variability in expression in non-human primates ($p=0.51$). Furthermore, when stratified by the presence of hominid specific transposon insertion, we observe decreased expression variability in in expression in non-hominid primates ($p<0.002$). We interpret these results to suggest that genes that tolerate TE insertions into CREs are not inherently tolerant to greater expression variability.

Supplemental methods

RNA-seq sample processing

We processed samples from all species in random batches of four in order to minimize batch effects. For each sample, 25 mg of frozen liver tissue was used to extract total RNA and genomic DNA, using QIAGEN AllPrep DNA/RNA/miRNA Universal Kit. Quality of total RNA was assessed by the RNA Integrity Number (RIN) using Agilent Bioanalyzer. All RNA samples had a RIN > 8. We used 4µg aliquots of total RNA to produce barcoded RNA sequencing libraries using the Illumina TruSeq Stranded mRNA kit. Libraries were pooled in two different pools based on barcode compatibility, and each pool was sequenced on two Illumina HiSeq2500 lanes, producing on an average of 42.1 million single end (SE) 100-bp reads per sample.

Detailed parameters used for RNA-seq alignment

We aligned all sequences that passed QC to the reference genomes from the Ensembl database (bushbaby: otoGar3; chimp: CHIMP2.1.4; humans: GRCh38; rhesus macaque: Mmul1; marmoset: C_jacchus3.2.1; mouse lemur: micMur1) using STAR v2.5 (Dobin et al., 2013), in 2-pass mode with the following parameters: --quantMode TranscriptomeSAM --outFilterMultimapNmax 10 --outFilterMismatchNmax 10 --outFilterMismatchNoverLmax 0.3 --alignIntronMin 21 --alignIntronMax 0 --alignMatesGapMax 0 --alignSJoverhangMin 5 --runThreadN 12 --twopassMode Basic --twopass1readsN 60000000 --sjdbOverhang 100. We filtered bam files based on alignment quality (q = 10) and sorted using Samtools v0.1.19 sort function (Li, 2009). We used the latest annotations for each species obtained from Ensembl to build reference indexes for the STAR alignment: Homo_sapiens.GRCh38.82.chr.gtf; Pan_troglodytes.CHIMP2.1.4.82.chr.gtf; Macaca_mulatta.MMUL_1.82.chr.gtf; Callithrix_jacchus.C_jacchus3.2.1.82.chr.gtf; Otolemur_garnettii.OtoGar3.82.gtf; Microcebus_murinus.micMur1.82.gtf (Aken et al., 2016).

ChIP-seq sample processing

We processed samples in six randomly assigned groups in order to minimize batch effects. For each sample, we cut 90 mg of frozen liver tissue into 1 mm3 pieces, washed the cut tissue samples with cold phosphate-buffered saline (PBS), and fixed with 1% formaldehyde for 5 minutes at room temperature. We prepared nuclei of each washed sample using the Covaris truChIP Tissue Chromatin Shearing Kit. Chromatin was then sheared for 16 minutes using a Covaris S220 Focused-ultrasonicator. We quantified shearing efficiency and chromatin concentration using Agilent Bioanalyzer High Sensitivity DNA Kit.

From each specimen, we kept aside a 0.5 μg aliquot of sheared chromatin to be used as input. We used two 5 μg aliquots of chromatin per sample to perform immunoprecipitation (IP) with antibodies directed at H3K27ac (ab4729) and H3K4me1 (ab8895) respectively. We performed each IP using 5 μg of antibody with an overnight incubation at 4°C as specified by the Magna ChIP A/G Chromatin Immunoprecipitation Kit protocol. After elution and protein-DNA crosslink reversal, we extracted DNA using Zymo Research ChIP DNA Clean & Concentrator kit, and quantified extracted DNA using Agilent High Sensitivity kit and Qubit 2.0. We used 5 to 15 ng of input and immunoprecipitated DNA to generate sequencing libraries using the NEBNext Ultra ChIPseq library kit, following protocols specified by the manufacturer. We assessed the quality of each constructed library using Agilent Bioanalyzer High Sensitivity DNA Kit and Kapa metrics. Libraries were multiplexed, pooled and sequenced on a total of 16 Illumina HiSeq2500 lanes, producing on an average of 40.6 million SE 100-bp reads per sample.

Peak calling QC

The following metrics were used for QC of peak calling: Fraction of Reads in Peaks (FRiP), Normalize Strand Correlation coefficient (NSC), Relative Strand Correlation coefficient (RSC), and ENCODE quality score. As recommended by the ENCODE consortium, we

selected a threshold of 1% as acceptable FRiP values. We computed the two strand correlation metrics (NSC, RSC) using Phantompeakqualtools (Landt et al., 2012). NSC ≥ 1.05 and RSC ≥ 0.8 were used as threshold for retaining samples (Table S1).

Parallelized reporter assay

We obtained a list of 334 putative 1-kb long CREs overlapping liver eQTLs from Brown and collaborators (Brown et al., 2013). This data included both enhancers (distance from TSS > 1Kb) and promoters (distance from TSS < 1kb). 276 CREs out of these 334 CREs overlapped our human ChIP-seq peaks (96 enhancers and 95 promoters; Table S6). Within each of the loci defined by the investigated liver eQTLs, we predicted a 1-kb CRE. These predicted CREs were amplified in individual PCRs performed on 120 pooled Yoruban HapMap DNA samples. PCR products from each reaction therefore represent a complex mixture of haplotypes. We inserted barcodes (hereafter, tags) consisting of a 160-bp oligo, including a randomized 20-bp unique barcode for each construct, into luciferase reporter vectors (pGL4.23 and pGL4.10), immediately downstream of the luciferase gene, after linearizing the vector with the XbaI restriction enzyme.

We pooled and cloned DNAs from each putative CRE into uniquely barcoded luciferase reporter vectors (pGL4.23 were used for enhancers and pGL4.10 for promoters), using the Gibson Assembly Kit (New England BioLabs). The CREs were specifically inserted upstream of the luciferase gene, after linearizing the vector with the restriction enzymes KpnI and XhoI. We then transfected the complex pool of CRE reporters into HepG2 cells in two replicates. 24 hours after transfection, we extracted total RNA, purified poly-A RNA, and produced cDNA that was used to amplify the tag, with the QIAGEN One Step RT-PCR Kit with primers that included Illumina adapters for sequencing. Tag libraries were pooled and sequenced on a single Illumina HiSeq2500 lane, producing single end (SE) 50-bp reads. We amplified the tags from the vector before the transfection and sequenced them in the same

pool with the tag-RNA libraries as a control for tag read counts.

In parallel, reporter tags were unambiguously associated with each specific CRE by sequence based sub-assembly. Briefly, we cut the luciferase gene from the vector by inverse PCR and then re-ligated the vector using the T4 Polynucleotide Kinase (PNK) + T4 ligase kit from NEB. In this way CREs and tags were flanking each other and CRE-tag complexes. The CRE-tag complexes were then PCR amplified using a reverse primer that included Illumina adapter for sequencing. Next, the CRE-tag PCR product was digested for 5 minutes at 55°C using Nexetera Tn5 Transposase (TDE1) in order to produce fragments of variable length (from ca. 150 bp to the entire length of the construct). When cutting the fragments, TDE1 also inserts an Illumina compatible adapter in proximity of the cutting site. We performed a PCR to enrich the libraries using the TDE1 inserted adapter as forward primer and the previously included Illumina adapter as reverse primer.

We pooled the two libraries (one for pGL4.10 and one for pGL4.23 constructs) and sequenced them on an Illumina MiSeq, producing paired-end (PE) reads (250 + 50 bp). After performing QC with FASTQC v0.11.3, we aligned the sub-assembly sequences to the human genome (GRCh37/hg19) using BWA mem and the bam files were sorted and indexed with Samtools v0.1.19. Finally, we produced a matrix listing all of the CRE-tag associations. Tags associated with more than one CRE were discarded and not used for further analyses. After attributing each tag to its uniquely associated CRE, we used sequence based tag counts (HiSeq reads), normalized by sequencing depth, to quantify the gene expression level driven by each CRE, and therefore its functionality as enhancer/promoter.

For each CRE, we used a count-based generalized linear model to quantify differential expression between RNA (after transfection) and DNA (before transfection), assuming a Poisson error function:

model= count~condition

where condition indicates that the read count comes either from RNA (replicates 1 and 2) or DNA-control.

In presence of a significant p-value, the model indicates a significant difference between the expression of the tags in the RNA samples compared to their DNA control. The effect size estimate was then used to infer whether the RNA samples were upregulated, hence showing significantly higher level of expression of the tags compared to their DNA controls, and therefore indicating that the CRE is a functional regulatory element.

Detection of orthologous regions for human peaks in each primate

We mapped orthologous sequences using all identified human consensus ChIP-seq peak regions in both H3K27ac and H3K4me1 experiments. We used the 40 Eutherian mammals Ensembl multiple sequence alignment (MSA) reference database with the following specifications: method_link_type:"EPO_LOW_COVERAGE"; species_set_name:"mammals" (Herrero et al., 2015; http://www.ensembl.org/info/genome/compara/analyses.html#epo). These alignments cover 88% of the human genome, 81% of the chimp genome, 85% of the macaque genome, 69% of the marmoset genome, 47% of the mouse lemur genome, and 57% of the bush baby genome. For orthologous sequence analysis, 500 bp up- and downstream regions were considered to be a part of the identified consensus peaks in all six species. We queried all regions directly from the reference database using the REST API (Yates et al., 2015) and downloaded respective coordinates for orthologous regions in each species as well as sequences for further analyses.

We independently queried each peak region +/- 500 bp. All orthologous sequences retained gaps generated by MSA. For differential histone modification binding analyses, mapped read counts from composite regions have been combined to represent analogous count matrices across all six species containing one unique count per queried region in each species. All orthologous sequences pulled from the references for downstream analysis contained only directly aligned sequences. All regions with no orthologs represented in the MSA reference were excluded from further analyses. All query results in .json format and

extracted sequences formatted for the MSA alignment as well as genomic position information are provided in the repository mentioned in the final section.

Correlation between human and marmoset read counts within orthologous regions

We assessed human and marmoset (i.e. the species with the smallest number of peaks called; Supplemental File S1) normalized read counts at the 39,710 orthologous CREs, after splitting them into two groups: 1) regions with overlapping peaks present in both marmoset and human, and 2) regions with a peak present only in human. Spearman's correlation (ρ) between human and marmoset normalized read depths was then computed for each the two groups.

Features associated with evolutionary conservation of CREs

We obtained publicly available data for DNase hypersensitivity sites (DHS) for 125 cell types (ENCODE Project Consortium, 2012) to estimate the number of cell types for which the CRE is functional. We estimated the correlation between the degree of conservation of CREs and the number of TFBSs by comparing our human consensus peaks with previously published HepG2 TF-binding profiles (ENCODE Project Consortium, 2012). We finally selected all genes within 10 kb distance from evolutionarily conserved CREs for gene set enrichment analysis using GOrilla software (Eden et al., 2007; Eden et al., 2009). All genes found within 10 kb of any of the 39,710 orthologous CREs are used as a background for the enrichment test.

Motif analysis

Genomic coordinates of orthologous regions were used to extract target sequences from the Ensembl references without MSA alignment gaps. All regions containing consensus peaks identified as human- and ape-specific and primate-conserved were used for the motif discovery and enrichment analysis, using the MEME Suite (Bailey et al., 2009, 2011). MEME-chip was used for known motif discovery and enrichment analysis using the Jaspar

database (Bailey et al., 2009). De novo motif identification was performed with AME (McLeay et al., 2010). Jaspar and Hocomoco (v10) databases were used as references to estimate similarities to known motifs. All motif discovery and enrichment analysis used default settings and parameters provided by the developers except for the maximum *de novo* motif discovery threshold (changed from 1 to 1000 for maximum threshold). Shuffled input sequences were used to estimate the background distribution of motifs.

Luciferase reporter assay validation of *GRIN3A* and *JARID2*

To test for species- or clade-specific regulatory activity, we compared activity of two predicted functional CREs with the empty pGL4.23 vector as a negative control. For *GRIN3A* we PCR amplified the CRE (Table S6), and cloned the fragment into pGL4.23 using the NEB Gibson Assembly Kit. The *JARID2* CRE was synthesized by GenScript and cloned into the same pGL4.23 vector. Cells were grown in DMEM high glucose (Gibco #11965084) supplemented with 10% fetal bovine serum (FBS) (GE Healthcare Life Sciences #SH3091003) containing antibiotic and antimycotic (Gibco #15240062) in a humidified incubator with 5% CO_2 at 37°C. HepG2 cells were seeded in 48-well CellBIND surface plates (Costar #3338) with 1.5 x 10^5 cells per well 24 h prior to transfection. Transfection complexes were formed using 800 ng of each construct with 1 µL of TransIT-LT1 transfection reagent (Mirus #MIR2304) and Opti-MEM (Gibso #31985070) in a total volume of 27 µL, incubated for 20 min and then added to cells. After transfection, cells were incubated for 24 h and were lysed in passive lysis buffer. To read firefly luciferase activity, 100 µL of LARII were added to 20 µL of cell lysate (from the dual-luciferase reporter assay system from Promega #E1910). We read Luminescence for 2 seconds per well on a 96-well compatible plate luminometer (ThermoFisher Luminoskan Ascent). The constructs were tested using three vector preparations in three to four technical transfection replicates (9 to 12 measurements for each construct). We normalized for transfection replicates effect using a linear model:

lm(log10(luciferase) ~ replicate + element.

Validation of the gene regulatory functionality of TE families

HepG2 cells were cultured in DMEM + GlutaMAX (Gibco) supplemented with 10% Fetal Bovine Serum (Gibco) and Normocin (InvivoGen). Transposable element constructs were built by synthesizing (GenScript) the Dfam (Hubley et al., 2016) consensus sequence for each element and cloning into the pGL3 Basic vector (Promega) with an added minimal promoter (pGL3 Basic[minP]). pGL3 BASIC[minP] with no insert was used as the negative expression control. pRL null (Promega) was the renilla control for transfection efficiency. TAP2 cloned into the pGL3 Basic[minP] was the positive control. Confluent HepG2 cells in opaque 96 well plates in 90ml of Opti-MEM (Gibco) were transfected according to the Lipofectamine p3000 protocol (Invitrogen) with 100 ng of the luciferase containing plasmid, 1 ng of pRL null, 0.3 ml of Lipofectamine 3000, and 0.2ml of p3000 reagent in 10 ml of Opti-MEM per well. The cells incubated in the transfection mixture for 24h hours then the media was then changed to the regular FBS containing media for an additional 24 hours. Dual Luciferase Reporter Assays (Promega) were started by incubating the cells for 15 mins in 20 ml of 1x passive lysis buffer. Luciferase and renilla expression were then measured using the Glomax multi+ detection system (Promega). Luciferase expression values of the transposable elements and TAP2 were standardized by the renilla expression values and background expression values as determined by pGL3-Basic expression. Enriched motifs were found by analyzing the Dfam (Hubley et al., 2016) consensus sequences of the TEs found to have a regulatory ability significantly different from the pGL3 Basic[minP] empty vector using the MEME Suite. TomTom (Gupta et al., 2007) was used to match binding site motifs in the Jaspar database to the enriched motifs found in our data.

Supplementary references

Aken, B.L., Ayling, S., Barrell, D., Clarke, L., Curwen, V., Fairley, S., Fernandez Banet, J., Billis, K., García Girón, C., Hourlier, T., et al. (2016). The Ensembl gene annotation system. Database 2016.

Bailey, T.L., Boden, M., Buske, F.A., Frith, M., Grant, C.E., Clementi, L., Ren, J., Li, W.W., and Noble, W.S. (2009). MEME SUITE: tools for motif discovery and searching. Nucleic Acids Research *37*, W202-208.

Brown, C.D., Mangravite, L., and Engelhardt B. (2013) Integrative Modeling of eQTLs and Cis-Regulatory Elements Suggests Mechanisms Underlying Cell Type Specificity of eQTLs. Plos Genetics http://dx.doi.org/10.1371/journal.pgen.1003649

Dobin, A., Davis, C.A., Schlesinger, F., Drenkow, J., Zaleski, C., Jha, S., Batut, P., Chaisson, M., and Gingeras, T.R. (2013). STAR: ultrafast universal RNA-seq aligner. Bioinformatics *29*(1), 15–21.

FANTOM Consortium and the RIKEN PMI and CLST (DGT) (2014). A promoter-level mammalian expression atlas. Nature *507*, 462–470.

Hubley, R., Finn, R.D., Clements, J., Eddy, S.R., Jones, T.A., Bao, W., Smit, A.F.A., and Wheeler, T.J. (2016). The Dfam database of repetitive DNA families. Nucleid Acids Research *44*, D81-D89.

Gupta, S, Stamatoyannopoulos, J.A., Bailey, T.L., and Noble, W.S. (2007). Quantifying similarity between motifs. Genome Biology *8*, R24.

Innocenti, F., Cooper, G.M., Stanaway, I.B., Gamazon, E.R., Smith, J.D., Mirkov, S., Ramirez, J., Liu, W., Lin, Y.S., Moloney, C., et al. (2011). Identification, Replication, and Functional Fine-Mapping of Expression Quantitative Trait Loci in Primary Human Liver

Tissue. PLoS Genet *7*, e1002078.

Liao, Y., Smyth, G.K., and Shi, W. (2014). featureCounts: an efficient general purpose program for assigning sequence reads to genomic features. Bioinformatics *30*, 923–930.

Li, (2009) samtools

McLeay, R.C., and Bailey, T.L. (2010). Motif Enrichment Analysis: a unified framework and an evaluation on ChIP data. BMC Bioinformatics *11*, 1–11.

Quinlan, A.R., and Hall, I.M. (2010). BEDTools: a flexible suite of utilities for comparing genomic features. Bioinformatics *26*, 841–842.

R Core Team (2016). R: A language and environment for statistical computing. R. Foundation for Statistical Computing, Vienna, Austria. URL https://www.R-project.org/.

Villar, D., Berthelot, C., Aldridge, S., Rayner, T.F., Lukk, M., Pignatelli, M., Park, T.J., Deaville, R., Erichsen, J.T., Jasinska, A.J., et al. Enhancer Evolution across 20 Mammalian Species. Cell *160*, 554–566.

Wickham., H. (2009). ggplot2: Elegant graphics for data analysis. Springer-Verlag New York, 2009.
