# Supplementary material for: Transposable elements are the primary source of novelty in primate gene regulation
Source: Genome Res. 2017 Oct;27(10):1623–33. doi: 10.1101/gr.218149.116 (PMC5630026; doi:10.1101/gr.218149.116)
Supplement: Supplemental Material [file supp_gr.218149.116_Supplemental_FILE_S5.pdf]

```
####RNASEQ

%%writefile 'RNASEQ.PROCESSING.sh'

#!/bin/bash

# Marco Trizzino

##########RNA-seq data processing

##FOR EACH INDIVIDUAL SAMPLE WE HAD SEQUENCES FROM TWO LANES:

#CALLING THE VARIABLES
echo $1 # Read RNA-seq sample lane 1
echo $2 # Read RNA-seq sample lane 2
echo $3 # reference genome
echo $4 # Sample output name
echo $5 # rsem reference

#PERFORMING QC OF THE SEQUENCES WITH FASTQC
fastqc $1.fastq -o /OUTPUT_PATH
fastqc $2.fastq -o /OUTPUT_PATH

#TRIMMING ADAPTERS FROM RNA-seq reads
trim_galore --cutadapt /cutadapt -stringency 5 -length 50 -o /
OUTPUT_PATH/ -q 20 $1.fastq $2.fastq

#STAR 2-PASS ALIGNMENT

STAR --limitGenomeGenerateRAM 240000000000 \\
--genomeDir $3 \\
--readFilesIn $1_trimmed.fq \\
--outSAMtype SAM \\
--quantMode TranscriptomeSAM \\
--outFilterMultimapNmax 10 \\
--outFilterMismatchNmax 10 \\
--outFilterMismatchNoverLmax 0.3 \\
--alignIntronMin 21 \\
--alignIntronMax 0 \\
--alignMatesGapMax 0 \\
--alignSJoverhangMin 5 \\
--runThreadN 12 \\
--twopassMode Basic \\
--twopass1readsN 60000000 \\
--sjdbOverhang 100 \\
--outFileNamePrefix $4_lane1.

STAR --limitGenomeGenerateRAM 240000000000 \\
--genomeDir $3 \\
--readFilesIn $2_trimmed.fq \\
```

```
--outSAMtype SAM \\
--quantMode TranscriptomeSAM \\
--outFilterMultimapNmax 10 \\
--outFilterMismatchNmax 10 \\
--outFilterMismatchNoverLmax 0.3 \\
--alignIntronMin 21 \\
--alignIntronMax 0 \\
--alignMatesGapMax 0 \\
--alignSJoverhangMin 5 \\
--runThreadN 12 \\
--twopassMode Basic \\
--twopass1readsN 60000000 \\
--sjdbOverhang 100 \\
--outFileNamePrefix $4_lane2.

mv $4_lane1.Aligned.out.sam $4_lane1_STAR.sam
mv $4_lane2.Aligned.out.sam $4_lane2_STAR.sam

#CONVERTING SAM IN BAM
samtools view -@ 8 -Sb $4_lane1_STAR.sam > $4_lane1_STAR.bam
samtools view -@ 8 -Sb $4_lane2_STAR.sam > $4_lane2_STAR.bam

#THE FOLLOWING COMMANDS SORT THE READS BASED ON CHROMOSOMES
COORDINATES
samtools sort -@ 8 -T $4_lane1_STAR.bam -O bam -o $4_lane1_STAR.bam
$4_lane1_STAR_srt.bam
samtools sort -@ 8 -T $4_lane2_STAR.bam -O bam -o $4_lane2_STAR.bam
$4_lane2_STAR_srt.bam

# merge BAM
samtools merge -f -@ 8 $4_mgd_STAR_srt.bam $4_lane1_STAR_srt.bam
$4_lane2_STAR_srt.bam

# MAPQ filter step
samtools view -@ 8 -q 10 -b $4_mgd_STAR_srt.bam >
$4_mgd_STAR_srt_q10.bam

#QUANTIFY GENE EXPRESSION WITH FEATURE COUNTS
featureCounts -T 16 -C -s 1 -t exon \\
-g gene_id -a /SPECIES_ENSEMBL_ANNOTATIONS/ \\
-o /OUTPUT_PATH/$4_feature_Counts.txt $4_mgd_STAR_srt_q10.bam
```

Orthologous gene identification

In [ ]:

```
%%writefile 'GENE.EXPRESSION.ANALYSIS.R'

# Marco Trizzino

################## FIND GENES ANNOTATED AS ORTHOLOGOUS IN THE SIX
STUDIED PRIMATES, USING BIOMART
```

```
#In R

source("http://bioconductor.org/biocLite.R")
biocLite("biomaRt") #363kb
library(biomaRt)

ensembl <-  useMart("ensembl", dataset = "hsapiens_gene_ensembl")
listDatasets(ensembl)
listAttributes(ensembl)
myFilter <- "ensembl_gene_id"
myAttributes <- c("ensembl_gene_id",
                  "ogarnettii_homolog_ensembl_gene",
                  "ptroglodytes_homolog_ensembl_gene",
                  "cjacchus_homolog_ensembl_gene",
                  "tbelangeri_homolog_ensembl_gene",
                  "mmurinus_homolog_ensembl_gene",
                  "mmulatta_homolog_ensembl_gene")
#The following step is slow:
GENEannot<- getBM(attributes =  myAttributes,
                  filters =  myFilter,
                  values =  rownames(Nreads),
                  mart = ensembl)
write.csv(file='ENSG_homologues.csv',GENEannot)
sum(GENEannot=="")
write.csv(file="ENSG_homologues_all.csv",
          GENEannot[GENEannot[,2]!="" & GENEannot[,3]!=""& GENEannot[,
4]!=""& GENEannot[,5]!=""& GENEannot[,6]!=""& GENEannot[,7]!="",],
          row.names=FALSE)


######################
#PREPARE MATRIX OF RNA-SEQ counts INCLUDING THE 10,243 ORTHOLOGOUS
GENEs FOR EACH SPECIMEN

####FOR EACH INDIVIDUAL DO AS FOLLOWS (EXAMPLE WITH CHIMP CH_4X0430:
Nreads <-read.delim(file="CH_4X0430_RNA_feature_Counts.txt", header=T,
row.names=NULL, sep="\t")
names(Nreads)[7]="CH_4X0430"
NreadsCOUNTS <- Nreads[,c(1,7)]
orthologs=read.csv("ENSG_homologues_all.csv",  header = TRUE)
index<-match(NreadsCOUNTS$Geneid,orthologs
$ptroglodytes_homolog_ensembl_gene)
NreadsCOUNTS$HumanID=orthologs$ensembl_gene_id[index]
Counts_ortho= NreadsCOUNTS[!is.na(NreadsCOUNTS$HumanID),]
write.csv(file="CH_4X0430_counts_ortho.csv", Counts_ortho,
row.names=FALSE)
#After processing all of the individual,
#merge the individual csv files in a single one
"GENE_COUNTS_ORTHOLOGOUS.csv":
#each file (= read counts for a give specimen) becomes a column
```

```
###IMPORTANT:
#Read counts need to be normalized by feature lengths before
performing differential expression
#Example for CH_4X0519
lengths= read.csv("feature_legths_per_species_per_gene.csv",
                  header=TRUE, row.names=NULL)
#open matrix with the feature lengths per each of the species for each
of the orthologous genes
lengths$CHIMP_factor <- lengths$HUMAN_lengths / lengths$CHIMP_lengths
counts=read.csv("GENE_COUNTS_ORTHOLOGOUS.csv", header = T)
counts$CH_4X0519_norm <- counts$CH_4X0519*counts$CHfactor



#### MATRIX WITH READ COUNTS FOR THE 10,683 ORTHOLOGOUS GENES,
#NORMALIZED BY FEATURE LENGTHS FOR THE SIX SPECIES IS TABLE S3



######################
#PERFORM DIFFERENTIAL EXPRESSION WITH DEseq2
#ON THE GENE COUNT MATRIX NORMALIZED BY FEATURE LENGTH (TABLE S3)

#Download DESeq2 from Bioconductor (only need to do the first time you
use DESeq2):
source("http://bioconductor.org/biocLite.R")
biocLite("DESeq2")

#Load DESeq2- Do this step every time you open R and need to run
DESeq2
library("DESeq2")

#Import your expression matrix- mine was a comma seperated values file
Nreads <-read.csv("GENE_COUNTS_ORTHOLOGOUS_NORM_FEAT_LENGTH.csv",
header=TRUE, row.names=1)
#CRITICAL: You need to use read countsfor DESeq2, not normalized data
like RPKMs (see manual).
#If read counts are not integers, they need to be round up to
integers:
Nreads_norm=round(Nreads,0)
#Omit any genes with zero counts across samples:
Nreads <- Nreads[rowSums(Nreads)!=0,]
#genes that have count=0 in only some of the genes need to be an
integer >0
Nreads=Nreads+1

#Now import a data frame that provides annotatios for the samples
#The rows of this correspond to your samples,
#column 1 has the name of the sample and column two the condition
```

```
(=species or species group)
#for humans vs other primates:
colData1 <-read.csv("colData_HSvsOTHER.csv", header=TRUE, row.names=1)
#HS VS ALL SPECIES
#for apes vs other primates:
colData2 <-read.csv("colData_APESvsALL.csv", header=TRUE, row.names=1)
#APES VS ALL SPECIES
#for all species vs all species pair-wise comparisons:
colData3 <-read.csv("colData_ALL_SPECIES.csv", header=TRUE,
row.names=1) #ALL SPECIES VS ALL SPECIES

###Now I want to look at 'HS' vs 'OTHERS' no fold change, FDR=0.05
#Perform DE analysis. You are telling it to compare by condition
dds <- DESeqDataSetFromMatrix(countData = Nreads, colData=colData1)
dds <- DESeq(dds)

res_HS_OTHER<-results(dds, contrast=c("condition","HS","OTHER"))
#Remove those genes for which a multiple correction p-value is "NA"
res_HS_OTHER_p=res_HS_OTHER[!is.na(res_HS_OTHER$padj), ]
#Only keep genes with corrected p-values below 0.05:
res_HS_OTHER_p=res_HS_OTHER_p[res_HS_OTHER_p$padj<0.05,]
#Save logfile
write.csv(as.data.frame(res_HS_OTHER_p), file = "HSVsOTHER.csv")
#Get the names of upregulated genes (HS>OTHER):
HighHS_OTHER=rownames(res_HS_OTHER_p[res_HS_OTHER_p
$log2FoldChange>0,])
write.csv(as.data.frame(HighHS_OTHER), file = "HighHS_OTHER.csv")
#Get the names of downregulated genes (HS<OTHER):
LowHS_OTHER=rownames(res_HS_OTHER_p[res_HS_OTHER_p$log2FoldChange<0,])
write.csv(as.data.frame(LowHS_OTHER), file = "LowHS_OTHER.csv")


###
#Now I want to look at 'APES' vs 'OTHERS' no fold change, FDR=0.05
dds <- DESeqDataSetFromMatrix(countData = Nreads, colData=colData2,
design= ~condition)
dds <- DESeq(dds)

res_APES_OTHER<-results(dds, contrast=c("condition","APES","OTHER"))
#Remove those genes for which a multiple correction p-value is "NA"
res_APES_OTHER_p=res_APES_OTHER[!is.na(res_APES_OTHER$padj), ]
#write.csv(as.data.frame(res_APES_OTHER_p), file =
"APESVsOTHER_ALL_P.csv")
#Only keep genes with corrected p-values below 0.05:
res_APES_OTHER_p=res_APES_OTHER_p[res_APES_OTHER_p$padj<0.05,]
#Get the names of upregulated genes (APES>OTHER):
HighAPES_OTHER=rownames(res_APES_OTHER_p[res_APES_OTHER_p
$log2FoldChange>0,])
write.csv(as.data.frame(HighAPES_OTHER), file = "HighAPES_OTHER.csv")
#Get the names of downregulated genes (APES<OTHER):
```

```
LowAPES_OTHER=rownames(res_APES_OTHER_p[res_APES_OTHER_p
$log2FoldChange<0,])
write.csv(as.data.frame(LowAPES_OTHER), file = "LowAPES_OTHER.csv")


###Now all of the possible human vs any other species pair-wise
combinations no fold change, FDR=0.05

dds <- DESeqDataSetFromMatrix(countData = Nreads, colData=colData3,
design= ~condition)
dds <- DESeq(dds)

# 'HS' vs 'BB'

res_HS_BB<-results(dds, contrast=c("condition","HS","BB"))
#Remove those genes for which a multiple correction p-value is "NA"
res_HS_BB_p=res_HS_BB[!is.na(res_HS_BB$padj), ]
#Only keep genes with corrected p-values below 0.05:
res_HS_BB_p=res_HS_BB_p[res_HS_BB_p$padj<0.05,]
#Get the names of upregulated genes (HS>BB):
HighHS_BB=rownames(res_HS_BB_p[res_HS_BB_p$log2FoldChange>0,])
write.csv(as.data.frame(HighHS_BB), file = "HighHS_BB.csv")
#Get the names of downregulated genes (HS<BB):
LowHS_BB=rownames(res_HS_BB_p[res_HS_BB_p$log2FoldChange<0,])
write.csv(as.data.frame(LowHS_BB), file = "LowHS_BB.csv")

#'HS' vs 'CH'

res_HS_CH<-results(dds, contrast=c("condition","HS","CH"))
#Remove those genes for which a multiple correction p-value is "NA"
res_HS_CH_p=res_HS_CH[!is.na(res_HS_CH$padj), ]
#Only keep genes with corrected p-values below 0.05:
res_HS_CH_p=res_HS_CH_p[res_HS_CH_p$padj<0.05,]
#Get the names of upregulated genes (HS>CH):
HighHS_CH=rownames(res_HS_CH_p[res_HS_CH_p$log2FoldChange>0,])
write.csv(as.data.frame(HighHS_CH), file = "HighHS_CH.csv")
#Get the names of downregulated genes (HS<CH):
LowHS_CH=rownames(res_HS_CH_p[res_HS_CH_p$log2FoldChange<0,])
write.csv(as.data.frame(LowHS_CH), file = "LowHS_CH.csv")

#'HS' vs 'RH'

res_HS_RH<-results(dds, contrast=c("condition","HS","RH"))
#Remove those genes for which a multiple correction p-value is "NA"
res_HS_RH_p=res_HS_RH[!is.na(res_HS_RH$padj), ]
#Only keep genes with corrected p-values below 0.05:
res_HS_RH_p=res_HS_RH_p[res_HS_RH_p$padj<0.05,]
#Get the names of upregulated genes (HS>RH):
HighHS_RH=rownames(res_HS_RH_p[res_HS_RH_p$log2FoldChange>0,])
write.csv(as.data.frame(HighHS_RH), file = "HighHS_RH.csv")
```

```
#Get the names of downregulated genes (HS<RH):
LowHS_RH=rownames(res_HS_RH_p[res_HS_RH_p$log2FoldChange<0,])
write.csv(as.data.frame(LowHS_RH), file = "LowHS_RH.csv")

#'HS' vs 'MS'

res_HS_MS<-results(dds, contrast=c("condition","HS","MS"))
#Remove those genes for whiMS a multiple correction p-value is "NA"
res_HS_MS_p=res_HS_MS[!is.na(res_HS_MS$padj), ]
#Only keep genes with corrected p-values below 0.05:
res_HS_MS_p=res_HS_MS_p[res_HS_MS_p$padj<0.05,]
#Get the names of upregulated genes (HS>MS):
HighHS_MS=rownames(res_HS_MS_p[res_HS_MS_p$log2FoldChange>0,])
write.csv(as.data.frame(HighHS_MS), file = "HighHS_MS.csv")
#Get the names of downregulated genes (HS<MS):
LowHS_MS=rownames(res_HS_MS_p[res_HS_MS_p$log2FoldChange<0,])
write.csv(as.data.frame(LowHS_MS), file = "LowHS_MS.csv")

#'HS' vs 'ML'

res_HS_ML<-results(dds, contrast=c("condition","HS","ML"))
#Remove those genes for which a multiple correction p-value is "NA"
res_HS_ML_p=res_HS_ML[!is.na(res_HS_ML$padj), ]
#Only keep genes with corrected p-values below 0.05:
res_HS_ML_p=res_HS_ML_p[res_HS_ML_p$padj<0.05,]
#Get the names of upregulated genes (HS>ML):
HighHS_ML=rownames(res_HS_ML_p[res_HS_ML_p$log2FoldChange>0,])
write.csv(as.data.frame(HighHS_ML), file = "HighHS_ML.csv")
#Get the names of downregulated genes (HS<ML):
LowHS_ML=rownames(res_HS_ML_p[res_HS_ML_p$log2FoldChange<0,])
write.csv(as.data.frame(LowHS_ML), file = "LowHS_ML.csv")

######################################################################
######################################################################
#####

#ChIP-seq

%%writefile 'run_fastqc_macs.py'

#!/usr/env/python

###
# YoSon
# usage: python run_fastqc_macs.py [samplelist.txt]
###

import os, subprocess, sys

wkdir = "primates/chipseq/"
```

```
scriptdir = wkdir + 'script/'
logdir = wkdir + 'logfiles/'

# make directories for the project

try:
    os.makedirs(wkdir)
except OSError:
    if not os.path.isdir(wkdir):
        raise

try:
    os.makedirs(scriptdir)
except OSError:
    if not os.path.isdir(scriptdir):
        raise

try:
    os.makedirs(logdir)
except OSError:
    if not os.path.isdir(logdir):
        raise


def mastercall(i):

    # write header for the job. adjust as necessary
    # for lsf scheduler

    master = scriptdir + str(i) + ".fastqc.macs.bsub"
    masterhandle=open(master, 'w')
    masterlist = []

    bsubfile = scriptdir + str(i) + ".fastqc.macs.bsub.sh"

    header=r"""#!/bin/bash
#BSUB -J %s.fdr5.fastqc.macs.bsub
#BSUB -o primates/chipseq/logfiles/%s.fdr1.fastqc.macs.bsub.o
#BSUB -e primates/chipseq/logfiles/%s.fdr1.fastqc.macs.bsub.e
#BSUB -n 8
#BSUB -R "span[ptile=8]"
#BSUB -M 50000
#BSUB -W 24:00

"""%(i, i, i)

    masterlist.append(header)
    for x in masterlist:
        masterhandle.write(x)
    masterhandle.write("sh " + bsubfile + " \n")
```

```
    masterhandle.close()


def bsubcall(i, fq1, fq2, fq3, ref, tfq1, tfq2, tfq3, out1, out2,
out3, chref):

    bsubfile = scriptdir + str(i) + ".fastqc.macs.bsub.sh"
    bsubhandle=open(bsubfile, 'w')
    bsublist = []

    # write script to submit

    bsubbatch="""#!/bin/bash

wkdir=primates/chipseq
sample='%s'
fq1=${wkdir}/fastq/test_pool/untrimmed/%s
fq2=${wkdir}/fastq/test_pool/untrimmed/%s
fq3=${wkdir}/fastq/test_pool/untrimmed/%s
#reference genome
ref=genomes/%s
tfqout=${wkdir}fastq/trimming_outputs/
tfq1='%s'
tfq2='%s'
tfq3='%s'
#h3k27ac
out1='%s'
#h3k4me1
out2='%s'
#input
out3='%s'
#reference chrom sizes
ref=chromosome_sizes/%s
fqout=primates/chipseq/fastq/fastqc_outputs

#----------------------------------------------------
# log all sample files and params passed

echo ${sample}
echo ${fq1} # Read 27ac fastq
echo ${fq2} # Read me1 fastq
echo ${fq3} # Read input fastq
echo ${ref} # reference genome
echo ${tfq1} # Trimmed 27ac fastq
echo ${tfq2} # Trimmed me1 fastq
echo ${tfq3} # Trimmed input fastq
echo ${out1} # Sample 27ac output name
echo ${out2} # Sample me1 output name
echo ${out3} # Sample input output name
echo ${chref} # chromosomes size
```

```
#---------------------------------------------------
# mkdir in case non-existent

mkdir -p ${wkdir}/fastq/test_pool/untrimmed/
mkdir -p ${wkdir}/fastq/trimming_outputs/
mkdir -p ${wkdir}/fastq/fastqc_outputs/

#---------------------------------------------------
# software used

FASTQC="bin/local/fastqc"
TRIM="bin/local/trim_galore"
CUTADAPT="bin/local/cutadapt"
BWA="bin/local/bwa"
#new samtools for sort, etc
SAMTOOLS1="bin/local/samtools"
#new samtools did not inherit functions such as rmdup, flagstats. path
to an old version
SAMTOOLS2="/opt/software/samtools/samtools-0.1.19/samtools"

#---------------------------------------------------
# qc
${FASTQC} ${fq1} ${fq2} ${fq3} -o ${fqout}

#---------------------------------------------------
# trimming adapters

# 27ac
${TRIM} --path_to_cutadapt ${CUTADAPT} -stringency 5 -length 35 -o $
{tfqout} -q 20 ${fq1}

# me1
${TRIM} --path_to_cutadapt ${CUTADAPT} -stringency 5 -length 35 -o $
{tfqout} -q 20 ${fq2}

# input
${TRIM} --path_to_cutadapt ${CUTADAPT} -stringency 5 -length 35 -o $
{tfqout} -q 20 ${fq3}


#---------------------------------------------------
# bwa alignment

# processing 27ac samples

###
#bwa-mem alignment

#bwa-mem alignment using 6 threads for all lanes (~average 16 lanes
```

```
per sample)
${BWA} mem -t 6 ${ref} ${tfq1} | ${SAMTOOLS2} view -Sb - > ${out1}
_mem.bam

# sort
${SAMTOOLS2} sort -@ 8 -T ${out1}_mem.bam -O bam -o ${out1}
_mem_srt.bam

#merge
${SAMTOOLS2} merge -f -@ 8 ${out2}_mgd_mem_sorted.bam *_mem_*.bam

#MAPQ = 10 filtering
${SAMTOOLS2} view -@ 8 -q 10 -b ${out2}_mgd_mem_sorted.bam > ${out2}
_mgd_mem_sorted_q10.bam

# remove dups
${SAMTOOLS2} rmdup -s ${out2}_mgd_mem_sorted_q10.bam ${out2}
_mgd_mem_sorted_rmdup.bam

#THE FOLLOWING COMMANDS RUN THE MAIN STATS FOR THE ALN SEQUENCES
BEFORE AND AFTER REMOVING PCR DUPLICATES
${SAMTOOLS2} flagstat ${out1}_mgd_mem_sorted.bam > ${out1}
_mgd_mem_sorted_stats.txt
${SAMTOOLS2} flagstat ${out1}_mgd_mem_sorted_rmdup.bam > ${out1}
_mgd_mem_sorted_rmdup_stats.txt


#---

# processing me1 samples

###
#bwa-mem alignment

#bwa-mem alignment using 6 threads for all lanes (~average 16 lanes
per sample)
${BWA} mem -t 6 ${ref} ${tfq2} | ${SAMTOOLS} view -Sb - > ${out2}
_mem.bam

# sort
${SAMTOOLS2} sort -@ 8 -T ${out2}_mem.bam -O bam -o ${out2}_mem.bam $
{out2}_mem_srt.bam

#merge
${SAMTOOLS2} merge -f -@ 8 ${out2}_mgd_mem_sorted.bam *_mem_*.bam


#MAPQ = 10 filtering
${SAMTOOLS2} view -@ 8 -q 10 -b ${out2}_mgd_mem_sorted.bam > ${out2}
_mgd_mem_sorted_q10.bam
```

```
# remove dups
${SAMTOOLS2} rmdup -s ${out2}_mgd_mem_sorted_q10.bam ${out2}
_mgd_mem_sorted_rmdup.bam

#THE FOLLOWING COMMANDS RUN THE MAIN STATS FOR THE ALN SEQUENCES
BEFORE AND AFTER REMOVING PCR DUPLICATES
${SAMTOOLS2} flagstat ${out2}_mgd_mem_sorted.bam > ${out2}
_mgd_mem_sorted_stats.txt
${SAMTOOLS2} flagstat ${out2}_mgd_mem_sorted_rmdup.bam > ${out2}
_mgd_mem_sorted_rmdup_stats.txt

###

#---
# processing input samples

###
#bwa-mem alignment

#bwa-mem alignment using 6 threads for all lanes (~average 16 lanes
per sample)
${BWA} mem -t 6 ${ref} ${tfq3} | ${SAMTOOLS2} view -Sb - > ${out3}
_mem.bam

# sort
${SAMTOOLS2} sort -@ 8 -T ${out3}_mem.bam -O bam -o ${out3}
_mem_srt.bam

#merge
${SAMTOOLS2} merge -f -@ 8 ${out3}_mem_srt.bam  *_mem_*.bam

#MAPQ = 10 filtering
${SAMTOOLS2} view -@ 8 -q 10 -b ${out3}_mgd_mem_srt.bam  > ${out3}
_mgd_mem_srt_q10.bam

# remove dups
${SAMTOOLS2} rmdup -s ${out3}_mgd_mem_sorted_q10.bam ${out3}
_mgd_mem_sorted_rmdup_q10.bam

#THE FOLLOWING COMMANDS RUN THE MAIN STATS FOR THE ALN SEQUENCES
BEFORE AND AFTER REMOVING PCR DUPLICATES
${SAMTOOLS2} flagstat ${out3}_mgd_mem_sorted.bam > ${out3}
_mgd_mem_sorted_stats.txt
${SAMTOOLS2} flagstat ${out3}_mgd_mem_sorted_rmdup_q10.bam > ${out3}
_mgd_mem_sorted_rmdup_q10_stats.txt


#---------------------------------------------------------------------
-
```

```
# peak calling

# 27ac peaks
# macs2
# q is for q-value, corresponding to FDR
# info here: https://github.com/taoliu/MACS/
macs2 callpeak -t ${out1}_mgd_mem_sorted_rmdup_q10.bam \\
-c ${out3}_mgd_mem_sorted_rmdup_q10.bam -f BAM -g hs -n ${out1}
_CSpeaks --no model --m 30 40 --extsize 147 -B -q 0.01

# me1 peaks
macs2 callpeak -t ${out2}_mgd_mem_sorted_rmdup_q10.bam \\
-c ${out3}_mgd_mem_sorted_rmdup_q10.bam -f BAM -g hs -n ${out2}
_CSpeaks --no model --m 30 40 --extsize 147 -B -q 0.01


#-------------------
#clean up

mkdir -p ${wkdir}/aln/flagstats/ ${wkdir}/peaks/

mv ${out1}_samse*.bam ${out2}_samse*.bam ${out3}_samse*.bam ${wkdir}/
aln/

mv ${out1}_samse_sorted_*stats.txt ${out2}_samse_sorted_*stats.txt $
{wkdir}/aln/flagstats/

mv ${out1}_CSpeaks_*.* ${out2}_CSpeaks_*.* ${wkdir}/peaks/


"""%(i, fq1, fq2, fq3, ref, tfq1, tfq2, tfq3, out1, out2, out3, chref)

    bsublist.append(bsubbatch)

    for x in bsublist:
        bsubhandle.write(x)
    bsubhandle.close()

for line in open('primates/chipseq/samples.txt', 'r').readlines():

    # example samples.txt
    #
    # Basil FGC1154_s_7_TCTCGCGC-GGCTCTGA.fastq \\
    # FGC1154_s_7_AGCGATAG-GGCTCTGA.fastq \\
    # FGC1154_s_7_ATTACTCG-AGGCGAAG.fastq \\
    # micMur1/micMur1bwaidx \\
    # FGC1154_s_7_TCTCGCGC-GGCTCTGA_trimmed.fq \\
    # FGC1154_s_7_AGCGATAG-GGCTCTGA_trimmed.fq \\
    # FGC1154_s_7_ATTACTCG-AGGCGAAG_trimmed.fq \\
    # ML_7022f_Basil_H3K27Ac \\
```

```
    # ML_7022f_Basil_H3K4me1 \\
    # ML_7022f_Basil_INPUT micMur1.chrom.sizes

    line = line.rstrip().split(' ')

    i = line[0]
    fq1 = line[1]
    fq2 = line[2]
    fq3 = line[3]
    ref = line[4]
    tfq1 = line[5]
    tfq2 = line[6]
    tfq3 = line[7]
    out1 = line[8]
    out2 = line[9]
    out3 = line[10]
    chref = line[11]

    mastercall(i)
    bsubcall(i, fq1, fq2, fq3, ref, tfq1, tfq2, tfq3, out1, out2,
out3, chref)

    master = scriptdir + "/" + str(i) + ".fastqc.macs.bsub"

    bsubline = "bsub < " + master
    os.system(bsubline)
```

ChIP-seq QC and strand correlation analyses

```
In [ ]:
%%writefile 'CHIPSEQ.QC.SH'

# Marco Trizzino

############################### PEAK CALLING QC
### FRACTION OF READS IN PEAKS (FRiP)


#CALLING THE VARIABLES
echo $1 # Chip-seq sample name

#Intersect the bam file (aln) with the bed file (peaks locations)
bedtools intersect -abam $1_mgd_mem_sorted_rmdup.bam \\
-b /$1_CSpeaks.narrowPeak -bed -c -f 0.20 > $1_intersect.bed

#Count how many reads you have in the peaks, using publicly available
perl script:
#(https://github.com/mel-astar/mel-ngs/blob/master/mel-chipseq/
chipseq-metrics/getCnt.pl)
perl getCnt.pl $1_intersect.bed > $1_FRiP.txt
```

```
### STRAND CORRELATION ANALYSES USING Phantompeakqualtools


#CALLING THE VARIABLES
echo $1 # bam Chip-seq sample name

Rscript --max-ppsize=500000 /phantompeakqualtools/run_spp.R \\
-c=$1_mgd_mem_sorted_rmdup.bam -savp -odir=/OUTPUT_PATH/ -out=
$1_run_spp.out
consensus peak calling

In [ ]:
%%writefile 'CONSENSUS.PEAK.CALLING.SH'

# Marco

############################ USING INDIVIDUAL THAT PASSED INDIVIDUAL
PEAK CALLING
#PERFORM SPECIES CONSENSUS PEAK CALLING
#!/bin/bash

#E.G. CONSENSUS H3K27Ac PEAKS
#CALLING THE VARIABLES
echo $1 # chip bam rep1
echo $2 # chip bam rep2
echo $3 # chip bam rep3
echo $4 # input bam
echo $5 # Sample output name

#CALLING PEAKS
macs2 callpeak -t $1 $2 $3 -c $4 -f BAM -g hs -n $5_CSpeaks --no model
--m 30 40 --extsize 147 -B -q 0.01
#YOU WILL GET SEVERAL OUTPUTS IN EXEL, BED AND BEDGRAPH FORMATS,
# SEE HERE FOR A DESCRIPTION: https://github.com/taoliu/MACS/
differential binding analyses

In [ ]:
%%writefile 'chipseq_analysis.r'

##################################
#USED 39 EUTHERIAN MAMMALS MULTISPECIES ALIGNMENT FROM ENSEMBL
# TO DEFINE REGIONS ORTHOLOGOUS TO HUMAN CONSENSUS PEAKS.
#AFTER DETECTING THE ORTHOLOGOUS (SEE YoSon's METHOD),
# MAP READ COUNTS FROM EACH SPECIES TO THE HUMAN ORTHOLOGOUS REGIONS
FOR H3K27Ac, H3K4me1 and iNPUT
### SEE SPECIFIC SCRIPT IN THE SUPPLEMENTARY MATHERIALS
### FINAL OUTPUT ARE THREE MATRIXES WITH ORTHOLOGOUS REGIONS AND
### H3K27Ac (MATRIX1), H3K4me1 (MATRIX2) and INPUT (MATRIX3)
RESPECTIVELY
```

```
###################################

###################### FOR BOTH H3K27Ac and H3K4me1 INDEPENDENTLY,
#PERFORM DIFFERENTIAL CHIP-SEQ BINDING ANALYSES WITH DEseq2
#ON THE ORTHOLOGOUS COUNT MATRIX NORMALIZED BY SEQUENCING DEPTH

#Download DESeq2 from Bioconductor (only need to do the first time you
use DESeq2):
source("http://bioconductor.org/biocLite.R")
biocLite("DESeq2")
library("DESeq2")


########## H3K27ac first

#Import your chipseq matrix
Nreads <-
read.csv("H3K27Ac_GENE_COUNTS_ORTHOLOGOUS_NORM_FEAT_LENGTH.csv",
header=TRUE, row.names=1)
#CRITICAL: You need to use read counts for DESeq2, not normalized data
like RPKMs (see manual).
#If read counts are not integers, they need to be round up to
integers:
Nreads_norm=round(Nreads,0)
#Omit any genes with zero counts across samples:
Nreads <- Nreads[rowSums(Nreads)!=0,]
#genes that have count=0 in only some of the genes need to be an
integer > 0
Nreads=Nreads+1

#Now import a data frame that provides annotatios for the samples
#The rows of this correspond to your samples, column 1 has the name of
the sample,
#column two the condition (=species or species group), column three is
the assay (CHIP or INPUT)
#for humans vs other primates:
colData1 <-read.csv("colData_HSvsOTHER.csv", header=TRUE, row.names=1)
#HS VS ALL SPECIES
#for apes vs other primates:
colData2 <-read.csv("colData_APESvsALL.csv", header=TRUE, row.names=1)
#APES VS ALL SPECIES
#for all species vs all species pair-wise comparisons:
colData3 <-read.csv("colData_ALL_SPECIES.csv", header=TRUE,
row.names=1) #ALL SPECIES VS ALL SPECIES


#ANALYSIS 1: HS Vs OTHERS (WALD TEST FOR INTERACTION ANALYSIS WITH
INPUT) no fold change, FDR=0.10
```

```
dds <- DESeqDataSetFromMatrix(countData = Nreads,
                              colData=colData1, design=~assay +
condition + assay:condition)
dds = DESeq(dds, test = "Wald")

res_HS_OTHER<-results(dds, contrast=c("condition","HS","OTHER"))
#Remove those genes for which a multiple correction p-value is "NA"
res_HS_OTHER_p=res_HS_OTHER[!is.na(res_HS_OTHER$padj), ]
#Only keep genes with corrected p-values below 0.10:
res_HS_OTHER_p=res_HS_OTHER_p[res_HS_OTHER_p$padj<0.10,]
#Save logfile
write.csv(as.data.frame(res_HS_OTHER_p), file =
"HSVsOTHER_Dbinding_27Ac.csv")
#Get the names of upbound enhancers (HS>other): they are human
specific enhancers
HighHS_OTHER=rownames(res_HS_OTHER_p[res_HS_OTHER_p
$log2FoldChange>0,])
write.csv(as.data.frame(HighHS_OTHER), file =
"human_specific_enh_27Ac.csv")


#ANALYSIS 2: APES Vs OTHERS (WALD TEST FOR INTERACTION ANALYSIS WITH
INPUT) no fold change, FDR=0.10

dds <- DESeqDataSetFromMatrix(countData = Nreads,
                              colData=colData2, design=~assay +
condition + assay:condition)
dds = DESeq(dds, test = "Wald")

res_APES_OTHER<-results(dds, contrast=c("condition","APES","OTHER"))
#Remove those genes for which a multiple correction p-value is "NA"
res_APES_OTHER_p=res_APES_OTHER[!is.na(res_APES_OTHER$padj), ]
#Only keep genes with corrected p-values below 0.10:
res_APES_OTHER_p=res_APES_OTHER_p[res_APES_OTHER_p$padj<0.10,]
#Save logfile
write.csv(as.data.frame(res_APES_OTHER_p), file =
"APESVsOTHER_Dbinding_27Ac.csv")
#Get the names of upbound enhancers (HS>other): they are apes specific
enh
HighAPES_OTHER=rownames(res_APES_OTHER_p[res_APES_OTHER_p
$log2FoldChange>0,])
write.csv(as.data.frame(HighAPES_OTHER), file =
"apes_specific_enh_27Ac.csv")


#Now all of the possible human vs any other species pair-wise
combinations no fold change, FDR=0.05

dds <- DESeqDataSetFromMatrix(countData = Nreads,
                              colData=colData3, design=~assay +
```

```
condition + assay:condition)
dds = DESeq(dds, test = "Wald")

# 'HS' vs 'CH'
res_HS_CH<-results(dds, contrast=c("condition","HS","CH"))
#Remove those genes for which a multiple correction p-value is "NA"
res_HS_CH_p=res_HS_CH[!is.na(res_HS_CH$padj), ]
#Only keep genes with corrected p-values below 0.10:
res_HS_CH_p=res_HS_CH_p[res_HS_CH_p$padj<0.10,]
#Save logfile
write.csv(as.data.frame(res_HS_CH_p), file =
"HSVsCH_Dbinding_27Ac.csv")

# 'HS' vs 'BB'
res_HS_BB<-results(dds, contrast=c("condition","HS","BB"))
#Remove those genes for which a multiple correction p-value is "NA"
res_HS_BB_p=res_HS_BB[!is.na(res_HS_BB$padj), ]
#Only keep genes with corrected p-values below 0.10:
res_HS_BB_p=res_HS_BB_p[res_HS_BB_p$padj<0.10,]
#Save logfile
write.csv(as.data.frame(res_HS_BB_p), file =
"HSVsBB_Dbinding_27Ac.csv")

#'HS' vs 'RH' no fold change
res_HS_RH<-results(dds, contrast=c("condition","HS","RH"))
#Remove those genes for which a multiple correction p-value is "NA"
res_HS_RH_p=res_HS_RH[!is.na(res_HS_RH$padj), ]
#Only keep genes with corrected p-values below 0.10:
res_HS_RH_p=res_HS_RH_p[res_HS_RH_p$padj<0.10,]
#Save logfile
write.csv(as.data.frame(res_HS_RH_p), file =
"HSVsRH_Dbinding_27Ac.csv")

#'HS' vs 'MS' no fold change
res_HS_MS<-results(dds, contrast=c("condition","HS","MS"))
#Remove those genes for which a multiple correction p-value is "NA"
res_HS_MS_p=res_HS_MS[!is.na(res_HS_MS$padj), ]
#Only keep genes with corrected p-values below 0.10:
res_HS_MS_p=res_HS_MS_p[res_HS_MS_p$padj<0.10,]
#Save logfile
write.csv(as.data.frame(res_HS_MS_p), file =
"HSVsMS_Dbinding_27Ac.csv")

#HS' vs 'ML' no fold change
res_HS_ML<-results(dds, contrast=c("condition","HS","ML"))
#Remove those genes for which a multiple correction p-value is "NA"
res_HS_ML_p=res_HS_ML[!is.na(res_HS_ML$padj), ]
#Only keep genes with corrected p-values below 0.10:
res_HS_ML_p=res_HS_ML_p[res_HS_ML_p$padj<0.10,]
#Save logfile
```

```
write.csv(as.data.frame(res_HS_ML_p), file =
"HSVsML_Dbinding_27Ac.csv")

########## H3K4me1 now

#Import your chipseq matrix
Nreads <-
read.csv("H3K4me1_GENE_COUNTS_ORTHOLOGOUS_NORM_FEAT_LENGTH.csv",
header=TRUE, row.names=1)
#CRITICAL: You need to use read counts for DESeq2, not normalized data
like RPKMs (see manual).
#If read counts are not integers, they need to be round up to
integers:
Nreads_norm=round(Nreads,0)
#Omit any genes with zero counts across samples:
Nreads <- Nreads[rowSums(Nreads)!=0,]
#genes that have count=0 in only some of the genes need to be an
integer > 0
Nreads=Nreads+1

#Now import a data frame that provides annotatios for the samples
#The rows of this correspond to your samples,
#column 1 has the name of the sample,
#column two the condition (=species or species group),
#column three is the assay (CHIP or INPUT)
#for humans vs other primates:
colData1 <-read.csv("colData_HSvsOTHER.csv", header=TRUE, row.names=1)
#HS VS ALL SPECIES
#for apes vs other primates:
colData2 <-read.csv("colData_APESvsALL.csv", header=TRUE, row.names=1)
#APES VS ALL SPECIES
#for all species vs all species pair-wise comparisons:
colData3 <-read.csv("colData_ALL_SPECIES.csv", header=TRUE,
row.names=1) #ALL SPECIES VS ALL SPECIES


#ANALYSIS 1: HS Vs OTHERS (WALD TEST FOR INTERACTION ANALYSIS WITH
INPUT) no fold change,
#FDR=0.10

dds <- DESeqDataSetFromMatrix(countData = Nreads,
                              colData=colData1, design=~assay +
condition + assay:condition)
dds = DESeq(dds, test = "Wald")

res_HS_OTHER<-results(dds, contrast=c("condition","HS","OTHER"))
#Remove those genes for which a multiple correction p-value is "NA"
res_HS_OTHER_p=res_HS_OTHER[!is.na(res_HS_OTHER$padj), ]
#Only keep genes with corrected p-values below 0.10:
res_HS_OTHER_p=res_HS_OTHER_p[res_HS_OTHER_p$padj<0.10,]
```

```
#Save logfile
write.csv(as.data.frame(res_HS_OTHER_p), file =
"HSVsOTHER_Dbinding_me1.csv")
#Get the names of upbound enhancers (HS>other): they are human
specific enhancers
HighHS_OTHER=rownames(res_HS_OTHER_p[res_HS_OTHER_p
$log2FoldChange>0,])
write.csv(as.data.frame(HighHS_OTHER), file =
"human_specific_enh_me1.csv")


#ANALYSIS 2: APES Vs OTHERS (WALD TEST FOR INTERACTION ANALYSIS WITH
INPUT) no fold change, FDR=0.10

dds <- DESeqDataSetFromMatrix(countData = Nreads,
                              colData=colData2, design=~assay +
condition + assay:condition)
dds = DESeq(dds, test = "Wald")

res_APES_OTHER<-results(dds, contrast=c("condition","APES","OTHER"))
#Remove those genes for which a multiple correction p-value is "NA"
res_APES_OTHER_p=res_APES_OTHER[!is.na(res_APES_OTHER$padj), ]
#Only keep genes with corrected p-values below 0.10:
res_APES_OTHER_p=res_APES_OTHER_p[res_APES_OTHER_p$padj<0.10,]
#Save logfile
write.csv(as.data.frame(res_APES_OTHER_p), file =
"APESVsOTHER_Dbinding_me1.csv")
#Get the names of upbound enhancers (HS>other): they are apes specific
enh
HighAPES_OTHER=rownames(res_APES_OTHER_p[res_APES_OTHER_p
$log2FoldChange>0,])
write.csv(as.data.frame(HighAPES_OTHER), file =
"apes_specific_enh_me1.csv")


#Now all of the possible human vs any other species pair-wise
combinations no fold change, FDR=0.05

dds <- DESeqDataSetFromMatrix(countData = Nreads,
                              colData=colData3, design=~assay +
condition + assay:condition)
dds = DESeq(dds, test = "Wald")

# 'HS' vs 'CH'
res_HS_CH<-results(dds, contrast=c("condition","HS","CH"))
#Remove those genes for which a multiple correction p-value is "NA"
res_HS_CH_p=res_HS_CH[!is.na(res_HS_CH$padj), ]
#Only keep genes with corrected p-values below 0.10:
res_HS_CH_p=res_HS_CH_p[res_HS_CH_p$padj<0.10,]
#Save logfile
```

```
write.csv(as.data.frame(res_HS_CH_p), file =
"HSVsCH_Dbinding_me1.csv")

# 'HS' vs 'BB'
res_HS_BB<-results(dds, contrast=c("condition","HS","BB"))
#Remove those genes for which a multiple correction p-value is "NA"
res_HS_BB_p=res_HS_BB[!is.na(res_HS_BB$padj), ]
#Only keep genes with corrected p-values below 0.10:
res_HS_BB_p=res_HS_BB_p[res_HS_BB_p$padj<0.10,]
#Save logfile
write.csv(as.data.frame(res_HS_BB_p), file =
"HSVsBB_Dbinding_me1.csv")

#'HS' vs 'RH' no fold change
res_HS_RH<-results(dds, contrast=c("condition","HS","RH"))
#Remove those genes for which a multiple correction p-value is "NA"
res_HS_RH_p=res_HS_RH[!is.na(res_HS_RH$padj), ]
#Only keep genes with corrected p-values below 0.10:
res_HS_RH_p=res_HS_RH_p[res_HS_RH_p$padj<0.10,]
#Save logfile
write.csv(as.data.frame(res_HS_RH_p), file =
"HSVsRH_Dbinding_me1.csv")

#'HS' vs 'MS' no fold change
res_HS_MS<-results(dds, contrast=c("condition","HS","MS"))
#Remove those genes for which a multiple correction p-value is "NA"
res_HS_MS_p=res_HS_MS[!is.na(res_HS_MS$padj), ]
#Only keep genes with corrected p-values below 0.10:
res_HS_MS_p=res_HS_MS_p[res_HS_MS_p$padj<0.10,]
#Save logfile
write.csv(as.data.frame(res_HS_MS_p), file =
"HSVsMS_Dbinding_me1.csv")

#HS' vs 'ML' no fold change
res_HS_ML<-results(dds, contrast=c("condition","HS","ML"))
#Remove those genes for which a multiple correction p-value is "NA"
res_HS_ML_p=res_HS_ML[!is.na(res_HS_ML$padj), ]
#Only keep genes with corrected p-values below 0.10:
res_HS_ML_p=res_HS_ML_p[res_HS_ML_p$padj<0.10,]
#Save logfile
write.csv(as.data.frame(res_HS_ML_p), file =
"HSVsML_Dbinding_me1.csv")


###################################################
DIFFERENTIAL BINDING ANALYSIS RESULTS ARE IN TABLES4

######################################################################
###############
```

```
#### PARSE DIFFERENTIAL CHIP-SEQ BINDING csv OUTPUTS
### FOR H3K27ac and H3K4me1 respectively get:
#- List of human specific elements
#- List of apes specific elements
#- List of conserved elements
#(= all of the elements that did not results as differentially
expressed in any of the comparisons)
#- List of other recently evolved elements
#(human specific + ape specific + other elements not conserved)

######################################################################
###############

# In R
# using human consensus H3K27ac and H3K4me1 peak files
# (narrowPeak bed file converted to txt format),
# using the name of the element (=name of the peak) as an index,
# associate peak coordinates to each element in the four file produced
in the previous step
# (conserved, human specific, apes specific, other recently evolved)

#H3K27ac
K27_peaks=read.table("CONSENSUS_H3K27Ac_narrowPeakfile.txt", header =
F)
human_specific=read.csv("human_specific_H3K27Ac_CREs.csv", header = F)
ape_specific=read.csv("ape_specific_H3K27Ac_CREs.csv", header = F)
other_recently_evolved=read.csv("other_recently_evolved_H3K27Ac_CREs.c
sv", header = F)
conserved=read.csv("other_conserved_H3K27Ac_.csv", header = F)

index<-match(K27_peaks$V4, human_specific$V1)
human_specific$chr=K27_peaks$V1[index]
human_specific$start=K27_peaks$V2[index]
human_specific$end=K27_peaks$end[index]
write.csv(as.data.frame(human_specific),
          file = 'human_specific_H3K27Ac_CREs_WITH_COORD.csv',
row.names = FALSE)

index<-match(K27_peaks$V4, ape_specific$V1)
ape_specific$chr=K27_peaks$V1[index]
ape_specific$start=K27_peaks$V2[index]
ape_specific$end=K27_peaks$end[index]
write.csv(as.data.frame(ape_specific),
          file = 'ape_specific_H3K27Ac_CREs_WITH_COORD.csv', row.names
= FALSE)

index<-match(K27_peaks$V4, other_recently_evolved$V1)
other_recently_evolved$chr=K27_peaks$V1[index]
other_recently_evolved$start=K27_peaks$V2[index]
other_recently_evolved$end=K27_peaks$end[index]
```

```
write.csv(as.data.frame(other_recently_evolved),
          file = 'other_recently_evolved_H3K27Ac_CREs_WITH_COORD.csv',
row.names = FALSE)

index<-match(K27_peaks$V4, conserved$V1)
conserved$chr=K27_peaks$V1[index]
conserved$start=K27_peaks$V2[index]
conserved$end=K27_peaks$end[index]
write.csv(as.data.frame(conserved),
          file = 'conserved_H3K27Ac_CREs_WITH_COORD.csv', row.names =
FALSE)

#H3K4me1
me1_peaks=read.table("CONSENSUS_H3K4me1_narrowPeakfile.txt", header =
F)
human_specific=read.csv("human_specific_H3K4me1_CREs.csv", header = F)
ape_specific=read.csv("ape_specific_H3K4me1_CREs.csv", header = F)
other_recently_evolved=read.csv("other_recently_evolved_H3K4me1_CREs.c
sv", header = F)
conserved=read.csv("other_conserved_H3K4me1_.csv", header = F)

index<-match(me1_peaks$V4, human_specific$V1)
human_specific$chr=me1_peaks$V1[index]
human_specific$start=me1_peaks$V2[index]
human_specific$end=me1_peaks$end[index]
write.csv(as.data.frame(human_specific),
          file = 'human_specific_H3K4me1_CREs_WITH_COORD.csv',
row.names = FALSE)

index<-match(me1_peaks$V4, ape_specific$V1)
ape_specific$chr=me1_peaks$V1[index]
ape_specific$start=me1_peaks$V2[index]
ape_specific$end=me1_peaks$end[index]
write.csv(as.data.frame(ape_specific),
          file = 'ape_specific_H3K4me1_CREs_WITH_COORD.csv', row.names
= FALSE)

index<-match(me1_peaks$V4, other_recently_evolved$V1)
other_recently_evolved$chr=me1_peaks$V1[index]
other_recently_evolved$start=me1_peaks$V2[index]
other_recently_evolved$end=me1_peaks$end[index]
write.csv(as.data.frame(other_recently_evolved),
          file = 'other_recently_evolved_H3K4me1_CREs_WITH_COORD.csv',
row.names = FALSE)

index<-match(me1_peaks$V4, conserved$V1)
conserved$chr=me1_peaks$V1[index]
conserved$start=me1_peaks$V2[index]
conserved$end=me1_peaks$end[index]
write.csv(as.data.frame(conserved),
```

```
         file = 'conserved_H3K4me1_CREs_WITH_COORD.csv', row.names =
FALSE)
```

functional analysis and annotations

In [ ]:

```
%%writefile 'functional_annotations.sh'


##########################
# For H3K27Ac and H3K4me1 independently, concatenate the four csv
files with coordinates and peak names (human specific, ape specific,
other recently evolved, conserved) in  single file

cat human_specific_H3K27Ac_CREs_WITH_COORD.csv
ape_specific_H3K27Ac_CREs_WITH_COORD.csv
other_recently_evolved_H3K27Ac_CREs_WITH_COORD.csv
conserved_H3K27Ac_CREs_WITH_COORD.csv > H3K27Ac_CREs_WITH_COORD.csv

cat human_specific_H3K4me1_CREs_WITH_COORD.csv
ape_specific_H3K4me1_CREs_WITH_COORD.csv
other_recently_evolved_H3K4me1_CREs_WITH_COORD.csv
conserved_H3K4me1_CREs_WITH_COORD.csv > H3K4me1_CREs_WITH_COORD.csv

# With a text editor convert the two concatenated files into two bed
files


###############################################################
## Now, for each of the two histone modifications we have a bed file
including: peak name, coordinates (chr, start, end) and conservation
status (human specific, ape specific, other recently evolved,
conserved)
## We use bedtools to check if some of the H3K27Ac peaks overlap some
of the H3K4me1 peaks (and vice-versa) for at list the 25% of their
length


bedtools intersect -a H3K4me1_ANNOTATED_PEAKS.bed -b
H3K27Ac_ANNOTATED_PEAKS.bed -f 0.25 -wa -wb >
H3K4me1_peaks_overlapping_H3K27Ac_peaks.txt

bedtools intersect -a H3K27Ac_ANNOTATED_PEAKS.bed -b
H3K4me1_ANNOTATED_PEAKS.bed -f 0.25 -wa -wb >
H3K27Ac_peaks_overlapping_H3K4me1_peaks.txt

## Concatenate H3K27Ac_ANNOTATED_PEAKS.bed and
H3K4me1_ANNOTATED_PEAKS.bed and using the information about the
overlapping peaks, include duplicated peaks only once
```

```
################################################################
## Annotate the matrix of unique not duplicated regulatory elements
(i.e. UNIQUE PEAKS) associating closest gene, closest gene typology
and distance from TSS of the closest gene

# First you need to convert the GTF file of the annotation to a bed
format using gtf2bed (from the Bedops suite)

gtf2bed GRCh38_ANNOTATIONS.gtf > GRCh38_ANNOTATIONS.bed

# Next from the gene annotations bed file (GRCh38_ANNOTATIONS.bed) you
need to extract the TSS coordinates (i.e. first base for + strand,
last base for  - strand).
# Final output will be bed file with TSS coordinates, strand and  gene
typology: 'GRCh38_TSSs.bed'

# Now associate a TSS to each cis-regulatory element:

bedtools closest -a UNIQUE_PEAKS_WITH_COORDINATES.bed -b
GRCh38_TSSs.bed > unique_peaks_with_coordinates_annotated.bed

## Now for each unique peak we have the following annotations: ID,
chr, start, end, additional histone mark (if duplicated peak),
coordinates of additional histone mark (if duplicated peak), closest
gene ID, closest gene typology, strand, distance from TSS



################################################################
#Use publicly available DHS data from ENCODE on 200+ cell types to
annotate the unique regulatory element matrix

bedtools intersect -a unique_peaks_with_coordinates_annotated.bed -b
ENCODE_DHS_DATA.bed -wa -wb >
unique_peaks_with_coordinates_annotated_including_cell_types.bed

################################################################
#Use publicly available data from ENCODE on TFBS on HepG2 cells to
annotate the unique regulatory element matrix

bedtools intersect -a
unique_peaks_with_coordinates_annotated_including_cell_types.bed -b
ENCODE_TFBS_DATA.bed -wa -wb >
unique_peaks_with_coordinates_annotated_including_cell_types_and_TFBS.
txt


## The matrix
'unique_peaks_with_coordinates_annotated_including_cell_types_and_TFBS
.txt' includes the following annotations: ID, chr, start, end,
```

```
additional histone mark (if duplicated peak), coordinates of
additional histone mark (if duplicated peak), closest gene ID, closest
gene typology, strand, distance from TSS, number of DHS cell types (if
any), number of TFBSs in HepG2 (if any)



##############################################################
## In R environment, using the peak ID as a index, annotate the matrix
with normalized read counts for each peak for each species

## Example with MARMOSET MS_32842 H3K27Ac counts

matrix=read.table('unique_peaks_with_coordinates_annotated_including_c
ell_types_and_TFBS.txt', header = T)
counts=read.csv('H3K27Ac_normalized_counts.csv', header=T)

index<-match(matrix$peak_ID, counts$peak_ID)
matrix$MS_32842_H3K27Ac=counts$MS_32842_H3K27Ac[index]
## Repeat this step for each individual and for each histone mark and
input

#### The final matrix is TABLES2 and includes the following
annotations: ID, chr, start, end, additional histone mark (if
duplicated peak), coordinates of additional histone mark (if
duplicated peak), closest gene ID, closest gene typology, strand,
distance from TSS, number of DHS cell types (if any), number of TFBSs
in HepG2 (if any), normalized read counts for each species for each
histone mark and input.

######################################
######################################
#TABLE_S2 is used for all the next downstream analyses
######################################
######################################
In [ ]:
%%bash

#MT

####################### Script for FIGURE 3

## Differentially modified CREs plot

# For each human centric pair-wise comparison quantify fraction of
differentially bound CREs
# You need to use outputs of DESeq2 analyses

cat 'HSVsCH_Dbinding.txt' | wc -l >
HS_CH_differentially_bound_numbers.txt
```

```
cat 'HSVsRH_Dbinding.txt' | wc -l >
HS_RH_differentially_bound_numbers.txt
cat 'HSVsMS_Dbinding.txt' | wc -l >
HS_MS_differentially_bound_numbers.txt
cat 'HSVsBB_Dbinding.txt' | wc -l >
HS_BB_differentially_bound_numbers.txt
cat 'HSVsML_Dbinding.txt' | wc -l >
HS_ML_differentially_bound_numbers.txt

# Concatenate the text files with the differential binding numbers
cat HS_CH_differentially_bound_numbers.txt
HS_RH_differentially_bound_numbers.txt
HS_MS_differentially_bound_numbers.txt
HS_BB_differentially_bound_numbers.txt
HS_ML_differentially_bound_numbers.txt >
primate_differential_binding_number.txt



## Differentially expressed genes plot

# For each human centric pair-wise comparison quantify fraction of
differentially bound CREs
# You need to use outputs of DESeq2 analyses

cat 'HSVsCH_DE.txt' | wc -l >
HS_CH_differentially_expressed_numbers.txt
cat 'HSVsRH_DE.txt' | wc -l >
HS_RH_differentially_expressed_numbers.txt
cat 'HSVsMS_DE.txt' | wc -l >
HS_MS_differentially_expressed_numbers.txt
cat 'HSVsBB_DE.txt' | wc -l >
HS_BB_differentially_expressed_numbers.txt
cat 'HSVsML_DE.txt' | wc -l >
HS_ML_differentially_expressed_numbers.txt

# Concatenate the text files with the differential binding numbers
cat HS_CH_differentially_expressed_numbers.txt
HS_RH_differentially_expressed_numbers.txt
HS_MS_differentially_expressed_numbers.txt
HS_BB_differentially_expressed_numbers.txt
HS_ML_differentially_expressed_numbers.txt >
primate_differential_binding_number.txt
```

Once all files are formatted, check out differential bindings (bar plot)

In [ ]:

```
%%R
```

```
# In R, Convert number to fractions
cres=read.table('primate_differential_binding_number.txt', header = F)
cres$V2=(cres$V1/47643)*100

# Now you need a column with species names
names = matrix(c(chimp, rhesus macaque, marmoset, bushbaby, mouse
lemur), nrow=5, ncol=1)

# Combine matrix with names with matrix with matrix with fractions
differential_binding_matrix=cbind(names$V1, cres$V2)
differential_binding_matrix$V1 <- factor(differential_binding_matrix
$V1, levels=unique(differential_binding_matrix$V1))

#BAR PLOT DIFFERENTIAL BINDING OVER SPECIES
ggplot(data=differential_binding_matrix, aes(x=V1, y=V2, fill=V1)) +
  geom_bar(stat="identity") +
  scale_fill_manual(values = c(
      "CHIMP"="#ff8c00",
      "RHESUS MACAQUE"="#db7093",
      "MARMOSET"="#4169e1",
      "BUSHBABY"="#00ced1",
      "MOUSE LEMUR"="#0000cd")) +
  labs(x="\nSPECIES",y="DIFFERENTIALLY BOUND CREs FRACTION (%)\n") +
  theme_minimal() +
  theme(axis.title=element_text(size=20),
axis.text=element_text(size=20))



# In R, convert number to fractions
DE=read.table('primate_differential_expressed_number.txt', header = F)
DE$V2=(DE$V1/47643)*100

# Now you need a column with species names
names = matrix(c(chimp, rhesus macaque, marmoset, bushbaby, mouse
lemur), nrow=5, ncol=1)

# Combine matrix with names with matrix with matrix with fractions
differential_expression_matrix=cbind(names$V1, cres$V2)
differential_expression_matrix$V1 <-
factor(differential_expression_matrix$V1,
levels=unique(differential_expression_matrix$V1))

#BAR PLOT DIFFERENTIAL EXPRESSION OVER SPECIES
ggplot(data=differential_expression_matrix, aes(x=V1, y=V2, fill=V1))
+
  geom_bar(stat="identity") +
  scale_fill_manual(values = c(
      "CHIMP"="#ff8c00",
      "RHESUS MACAQUE"="#db7093",
```

```
      "MARMOSET"="#4169e1",
      "BUSHBABY"="#00ced1",
      "MOUSE LEMUR"="#0000cd")) +
  labs(x="\nSPECIES",y="DIFFERENTIALLY EXPRESSED GENES FRACTION (%)
\n") +
  theme_minimal() +
  theme(axis.title=element_text(size=20),
axis.text=element_text(size=20))
```

In [ ]:

```
%%bash


###################### Script for FIGURE 4

######### Gene typologies: Figure 4a

## From TABLES2.txt, extract conserved and recently evolved CREs
cat TABLE2.txt | grep "conserved"  > conserved_CREs.txt
cat TABLE2.txt | grep -e "human_specific" -e "apes_specific" -e
"other_recently_evolved"  > recently_evolved_CREs.txt

# Count conserved and recently evolved CREs associated to protein
coding genes
cat conserved_CREs.txt | grep "protein" | wc -l >
conserved_protein_coding_numbers.txt
cat recently evolved_CREs.txt | grep "protein" | wc -l >
recently_evolved_protein_coding_numbers.txt

# Count conserved and recently evolved CREs associated to lincRNAs
cat conserved_CREs.txt | grep "lincRNA" | wc -l >
conserved_lincRNA_numbers.txt
cat recently evolved_CREs.txt | grep "lincRNA" | wc -l >
recently_evolved_lincRNA_numbers.txt

# Count conserved and recently evolved CREs associated to pseudogenes
cat conserved_CREs.txt | grep "pseudogene" | wc -l >
conserved_pseudogene_numbers.txt
cat recently evolved_CREs.txt | grep "pseudogene" | wc -l >
recently_pseudogene_lincRNA_numbers.txt

# Concatenate all of the conserved, adding also the total number of
conserved and the total number of labile derived from Figure 2
analyses (conserved_number.txt and recently_evolved_number.txt files)
cat conserved_number.txt recently_evolved_numbers. txt
conserved_protein_coding_numbers.txt
recently_evolved_protein_coding_numbers.txt
conserved_lincRNA_numbers.txt recently_evolved_lincRNA_numbers.txt
conserved_pseudogene_numbers.txt
recently_evolved_pseudogene_numbers.txt > gene_types_N.txt
```

In [ ]:

```
%%R

#MT

## In R environment
gene_types = read.table('gene_types_N.txt', header = F)

# Convert numbers to fractions
gene_types$V2=(gene_types $V1/47643)*100

# Build a matrix with gene type names
gene_type_names = matrix(c(all_conserved, all_recently_evolved,
protein_coding_conserved, protein_coding_recently_evolved,
lincRNA_conserved, lincRNA_recently_evolved,
pseudogene_conserved_pseudogene_reently_evolved), nrow=8, ncol=1)

# Bind the three matrixes
matrix = cbind(gene_type_names$V1, gene_types$V2)

# PLOT them
ggplot(data=matrix, aes(x=V1, y=V2, fill=V1)) +
geom_bar(stat="identity") +  scale_fill_manual(values = c(
  "all_conserved"="#0073ff",
  "all_recently_evolved"="#ff0d00",
  "protein_coding_conserved"="#0073ff",
  "protein_coding_recently_evolved"="#ff0d00",
  "lincRNA_conserved"="#0073ff",
  "lincRNA_recently_evolved" ="#ff0d00",
  "Pseudogenes_conserved"="#0073ff",
  "Pseudogenes_recently_evolved" ="#ff0d00"))+
  labs(x="\nGENE CATEGORY",y="FRACTION OF THE GENES ASSOCIATED CREs
(%)\n")+
  scale_x_discrete(limits=c("all_conserved", "all_recently_evolved",
"","protein_coding_conserved","protein_coding_recently_evolved","","li
ncRNA_conserved","lincRNA_recently_evolved","","Pseudogenes_conserved"
, "Pseudogenes_recently_evolved"))+
  theme_minimal()+
  theme(axis.title=element_text(size=16),
axis.text=element_text(size=16))+
  theme(axis.text.x = element_text(angle = 90, hjust = 1))
In [ ]:
%%bash

#MT

######## TSS distances

# Break down the conserved and labile CREs by species, including the
annotations on the distance from the closest TSS from Table S2
```

```
#Example: CHIMP

# Concatenate the differential binding analyses outputs from the human
Vs chimp comparison in H3K27Ac and H3K4me1
cat HSVsCH_Dbinding_27Ac.csv HSVsCH_Dbinding_me1.csv >
HSVsCH_Dbinding_all.csv
cat HSVsRH_Dbinding_27Ac.csv HSVsRH_Dbinding_me1.csv >
HSVsRH_Dbinding_all.csv
cat HSVsMS_Dbinding_27Ac.csv HSVsMS_Dbinding_me1.csv >
HSVsMS_Dbinding_all.csv
cat HSVsBB_Dbinding_27Ac.csv HSVsBB_Dbinding_me1.csv >
HSVsBB_Dbinding_all.csv
cat HSVsML_Dbinding_27Ac.csv HSVsML_Dbinding_me1.csv >
HSVsML_Dbinding_all.csv
In [ ]:
%%R

#MT


# In R

# For each species:

# Chimp
chimp_diff=read.csv('HSVsCH_Dbinding_all.csv', header=F)
All_CREs=read.table(TABLES2.txt, header=T)
index<-match(All_CREs$CRE_ID,chimp_diff$V1)
All_CREs$chimp=chimp_diff$V1[index]
labile_chimp=na.omit(all_CREs)
conserved_chimp=all_CREs[is.na(all_CREs$chimp),]
write.csv(as.data.frame(labile_chimp), file =
"Labile_chimp_distances.csv", row.names = FALSE)
write.csv(as.data.frame(conserved_chimp), file =
"Conserved_chimp_distances.csv", row.names = FALSE)

# Rhesus macaque
rhesus_diff=read.csv('HSVsRH_Dbinding_all.csv', header=F)
All_CREs=read.table(TABLES2.txt, header=T)
index<-match(All_CREs$CRE_ID,rhesus_diff$V1)
All_CREs$rhesus=rhesus_diff$V1[index]
labile_rhesus=na.omit(all_CREs)
conserved_rhesus=all_CREs[is.na(all_CREs$rhesus),]
write.csv(as.data.frame(labile_rhesus), file =
"Labile_rhesus_distances.csv", row.names = FALSE)
write.csv(as.data.frame(conserved_rhesus), file =
"Conserved_rhesus_distances.csv", row.names = FALSE)

# Marmoset
marmoset_diff=read.csv('HSVsMS_Dbinding_all.csv', header=F)
```

```
All_CREs=read.table(TABLES2.txt, header=T)
index<-match(All_CREs$CRE_ID,marmoset_diff$V1)
All_CREs$marmoset=marmoset_diff$V1[index]
labile_marmoset=na.omit(all_CREs)
conserved_marmoset=all_CREs[is.na(all_CREs$marmoset),]
write.csv(as.data.frame(labile_marmoset), file =
"Labile_marmoset_distances.csv", row.names = FALSE)
write.csv(as.data.frame(conserved_marmoset), file =
"Conserved_marmoset_distances.csv", row.names = FALSE)

# Bushbaby
bushbaby_diff=read.csv('HSVsBB_Dbinding_all.csv', header=F)
All_CREs=read.table(TABLES2.txt, header=T)
index<-match(All_CREs$CRE_ID,bushbaby_diff$V1)
All_CREs$bushbaby=bushbaby_diff$V1[index]
labile_bushbaby=na.omit(all_CREs)
conserved_bushbaby=all_CREs[is.na(all_CREs$bushbaby),]
write.csv(as.data.frame(labile_bushbaby), file =
"Labile_bushbaby_distances.csv", row.names = FALSE)
write.csv(as.data.frame(conserved_bushbaby), file =
"Conserved_bushbaby_distances.csv", row.names = FALSE)

# Mouse lemur
mouse_lemur_diff=read.csv('HSVsML_Dbinding_all.csv', header=F)
All_CREs=read.table(TABLES2.txt, header=T)
index<-match(All_CREs$CRE_ID,mouse_lemur_diff$V1)
All_CREs$mouse_lemur=mouse_lemur_diff$V1[index]
labile_mouse_lemur=na.omit(all_CREs)
conserved_mouse_lemur=all_CREs[is.na(all_CREs$mouse_lemur),]
write.csv(as.data.frame(labile_mouse_lemur), file =
"Labile_mouse_lemur_distances.csv", row.names = FALSE)
write.csv(as.data.frame(conserved_mouse_lemur), file =
"Conserved_mouse_lemur_distances.csv", row.names = FALSE)


## In each of the produced species dataframe, compute the fraction of
conserved CREs within each of the following 5 kb windows: 0 kb, 0.1-5,
5-10, 10-15, 15-20, 20-25, 25-30, > 30


# chimp
conserved=read.csv('Conserved_chimp_distances.csv', header = F)
labile=read.csv('Labile_chimp_distances.csv', header = F)

chimp_conserved0kb = (sum( conserved$V1 == 0 )/(sum( conserved$V1 ==
0 ) + sum( labile$V1 == 0  ))) *100
chimp_conserved0_5kb = (sum( 0 %<% conserved$V1 %<% 5 )/(sum( 0 %<%
conserved$V1 %<% 5 ) + sum( 0 %<% labile$V1 %<% 5 ))) *100
chimp_conserved5_10kb = (sum( 5 %<% conserved$V1 %<% 10 )/(sum( 5 %<%
conserved$V1 %<% 10 ) + sum( 5 %<% labile$V1 %<% 10 ))) *100
```

```
chimp_conserved10_15kb = (sum( 10 %<% conserved$V1 %<% 15 )/(sum( 10
%<% conserved$V1 %<% 15 ) + sum( 10 %<% labile$V1 %<% 15 ))) *100
chimp_conserved15_20kb = (sum( 15 %<% conserved$V1 %<% 20 )/(sum( 15
%<% conserved$V1 %<% 20 ) + sum( 15 %<% labile$V1 %<% 20 ))) *100
chimp_conserved20_25kb = (sum( 20 %<% conserved$V1 %<% 25 )/(sum( 20
%<% conserved$V1 %<% 25 ) + sum( 20 %<% labile$V1 %<% 25 ))) *100
chimp_conserved25_30kb = (sum( 25 %<% conserved$V1 %<% 30 )/(sum( 25
%<% conserved$V1 %<% 30 ) + sum( 25 %<% labile$V1 %<% 30 ))) *100
chimp_conserved>_30kb = (conserved$V1 %>% 30 )/(sum( conserved$V1 %>%
30  ) + sum( labile$V1 %>% 30  ))) *100


# rhesus
conserved=read.csv('Conserved_rhesus_distances.csv', header = F)
labile=read.csv('Labile_rhesus_distances.csv', header = F)

rhesus_conserved0kb = (sum( conserved$V1 == 0 )/(sum( conserved$V1 ==
0 ) + sum( labile$V1 == 0  ))) *100
rhesus_conserved0_5kb = (sum( 0 %<% conserved$V1 %<% 5 )/(sum( 0 %<%
conserved$V1 %<% 5 ) + sum( 0 %<% labile$V1 %<% 5 ))) *100
rhesus_conserved5_10kb = (sum( 5 %<% conserved$V1 %<% 10 )/(sum( 5 %<%
conserved$V1 %<% 10 ) + sum( 5 %<% labile$V1 %<% 10 ))) *100
rhesus_conserved10_15kb = (sum( 10 %<% conserved$V1 %<% 15 )/(sum( 10
%<% conserved$V1 %<% 15 ) + sum( 10 %<% labile$V1 %<% 15 ))) *100
rhesus_conserved15_20kb = (sum( 15 %<% conserved$V1 %<% 20 )/(sum( 15
%<% conserved$V1 %<% 20 ) + sum( 15 %<% labile$V1 %<% 20 ))) *100
rhesus_conserved20_25kb = (sum( 20 %<% conserved$V1 %<% 25 )/(sum( 20
%<% conserved$V1 %<% 25 ) + sum( 20 %<% labile$V1 %<% 25 ))) *100
rhesus_conserved25_30kb = (sum( 25 %<% conserved$V1 %<% 30 )/(sum( 25
%<% conserved$V1 %<% 30 ) + sum( 25 %<% labile$V1 %<% 30 ))) *100
rhesus_conserved>_30kb = (conserved$V1 %>% 30 )/(sum( conserved$V1 %>%
30  ) + sum( labile$V1 %>% 30  ))) *100


# marmoset
conserved=read.csv('Conserved_marmoset_distances.csv', header = F)
labile=read.csv('Labile_marmoset_distances.csv', header = F)

marmoset_conserved0kb = (sum( conserved$V1 == 0 )/(sum( conserved$V1
== 0 ) + sum( labile$V1 == 0  ))) *100
marmoset_conserved0_5kb = (sum( 0 %<% conserved$V1 %<% 5 )/(sum( 0 %<%
conserved$V1 %<% 5 ) + sum( 0 %<% labile$V1 %<% 5 ))) *100
marmoset_conserved5_10kb = (sum( 5 %<% conserved$V1 %<% 10 )/(sum( 5
%<% conserved$V1 %<% 10 ) + sum( 5 %<% labile$V1 %<% 10 ))) *100
marmoset_conserved10_15kb = (sum( 10 %<% conserved$V1 %<% 15 )/
(sum( 10 %<% conserved$V1 %<% 15 ) + sum( 10 %<% labile$V1 %<% 15 )))
*100
marmoset_conserved15_20kb = (sum( 15 %<% conserved$V1 %<% 20 )/
(sum( 15 %<% conserved$V1 %<% 20 ) + sum( 15 %<% labile$V1 %<% 20 )))
*100
```

```
marmoset_conserved20_25kb = (sum( 20 %<% conserved$V1 %<% 25 )/
(sum( 20 %<% conserved$V1 %<% 25 ) + sum( 20 %<% labile$V1 %<% 25 )))
*100
marmoset_conserved25_30kb = (sum( 25 %<% conserved$V1 %<% 30 )/
(sum( 25 %<% conserved$V1 %<% 30 ) + sum( 25 %<% labile$V1 %<% 30 )))
*100
marmoset_conserved>_30kb = (conserved$V1 %>% 30 )/(sum( conserved$V1
%>% 30  ) + sum( labile$V1 %>% 30  ))) *100


# bushbaby
conserved=read.csv('Conserved_bushbaby_distances.csv', header = F)
labile=read.csv('Labile_bushbaby_distances.csv', header = F)

bushbaby_conserved0kb = (sum( conserved$V1 == 0 )/(sum( conserved$V1
== 0 ) + sum( labile$V1 == 0  ))) *100
bushbaby_conserved0_5kb = (sum( 0 %<% conserved$V1 %<% 5 )/(sum( 0 %<%
conserved$V1 %<% 5 ) + sum( 0 %<% labile$V1 %<% 5 ))) *100
bushbaby_conserved5_10kb = (sum( 5 %<% conserved$V1 %<% 10 )/(sum( 5
%<% conserved$V1 %<% 10 ) + sum( 5 %<% labile$V1 %<% 10 ))) *100
bushbaby_conserved10_15kb = (sum( 10 %<% conserved$V1 %<% 15 )/
(sum( 10 %<% conserved$V1 %<% 15 ) + sum( 10 %<% labile$V1 %<% 15 )))
*100
bushbaby_conserved15_20kb = (sum( 15 %<% conserved$V1 %<% 20 )/
(sum( 15 %<% conserved$V1 %<% 20 ) + sum( 15 %<% labile$V1 %<% 20 )))
*100
bushbaby_conserved20_25kb = (sum( 20 %<% conserved$V1 %<% 25 )/
(sum( 20 %<% conserved$V1 %<% 25 ) + sum( 20 %<% labile$V1 %<% 25 )))
*100
bushbaby_conserved25_30kb = (sum( 25 %<% conserved$V1 %<% 30 )/
(sum( 25 %<% conserved$V1 %<% 30 ) + sum( 25 %<% labile$V1 %<% 30 )))
*100
bushbaby_conserved>_30kb = (conserved$V1 %>% 30 )/(sum( conserved$V1
%>% 30  ) + sum( labile$V1 %>% 30  ))) *100


# mouse lemur
conserved=read.csv('Conserved_mouse_lemur_distances.csv', header = F)
labile=read.csv('Labile_mouse_lemur_distances.csv', header = F)

mouse_lemur_conserved0kb = (sum( conserved$V1 == 0 )/(sum( conserved
$V1 == 0 ) + sum( labile$V1 == 0  ))) *100
mouse_lemur_conserved0_5kb = (sum( 0 %<% conserved$V1 %<% 5 )/(sum( 0
%<% conserved$V1 %<% 5 ) + sum( 0 %<% labile$V1 %<% 5 ))) *100
mouse_lemur_conserved5_10kb = (sum( 5 %<% conserved$V1 %<% 10 )/
(sum( 5 %<% conserved$V1 %<% 10 ) + sum( 5 %<% labile$V1 %<% 10 )))
*100
mouse_lemur_conserved10_15kb = (sum( 10 %<% conserved$V1 %<% 15 )/
(sum( 10 %<% conserved$V1 %<% 15 ) + sum( 10 %<% labile$V1 %<% 15 )))
*100
```

```
mouse_lemur_conserved15_20kb = (sum( 15 %<% conserved$V1 %<% 20 )/
(sum( 15 %<% conserved$V1 %<% 20 ) + sum( 15 %<% labile$V1 %<% 20 )))
*100
mouse_lemur_conserved20_25kb = (sum( 20 %<% conserved$V1 %<% 25 )/
(sum( 20 %<% conserved$V1 %<% 25 ) + sum( 20 %<% labile$V1 %<% 25 )))
*100
mouse_lemur_conserved25_30kb = (sum( 25 %<% conserved$V1 %<% 30 )/
(sum( 25 %<% conserved$V1 %<% 30 ) + sum( 25 %<% labile$V1 %<% 30 )))
*100
mouse_lemur_conserved>_30kb = (conserved$V1 %>% 30 )/(sum( conserved
$V1 %>% 30  ) + sum( labile$V1 %>% 30  ))) *100


## Report them in the next single dataframe
'TSS_5Kb_WINDOWS_with_conserved_fractions.csv'


#PLOT CONSERVATION FRACTION BY DISTANCE PER SPECIES
TSS_DISTANCE =
read.csv("TSS_5Kb_WINDOWS_with_conserved_fractions.csv", header = T)
TSS_DISTANCE$Distance_from_TSS <- factor(TSS_DISTANCE
$Distance_from_TSS, levels=unique(TSS_DISTANCE$Distance_from_TSS))

ggplot()+

geom_line(aes_string(x='Distance_from_TSS',y='Fraction_conserved_chimp
'), color='#ff8c00', data=TSS_DISTANCE, group =1, size = 2)+

geom_point(aes_string(x='Distance_from_TSS',y='Fraction_conserved_chim
p'), color='#ff8c00',shape=19, size=5, data=TSS_DISTANCE)+

geom_line(aes_string(x='Distance_from_TSS',y='Fraction_conserved_rhesu
s'), color='#db7093', data=TSS_DISTANCE, group =1, size = 2)+

geom_point(aes_string(x='Distance_from_TSS',y='Fraction_conserved_rhes
us'), color='#db7093',shape=19, size=5, data=TSS_DISTANCE)+

geom_line(aes_string(x='Distance_from_TSS',y='Fraction_conserved_marmo
set'), color='#4169e1', data=TSS_DISTANCE, group =1, size = 2)+

geom_point(aes_string(x='Distance_from_TSS',y='Fraction_conserved_marm
oset'), color='#4169e1',shape=19, size=5, data=TSS_DISTANCE)+

geom_line(aes_string(x='Distance_from_TSS',y='Fraction_conserved_bushb
aby'), color='#00ced1', data=TSS_DISTANCE, group =1, size = 2)+

geom_point(aes_string(x='Distance_from_TSS',y='Fraction_conserved_bush
baby'), color='#00ced1',shape=19, size=5, data=TSS_DISTANCE)+

geom_line(aes_string(x='Distance_from_TSS',y='Fraction_conserved_mouse
```

```
_lemur'), color='#0000cd', data=TSS_DISTANCE, group =1, size = 2)+

geom_point(aes_string(x='Distance_from_TSS',y='Fraction_conserved_mous
e_lemur'), color='#0000cd',shape=19, size=5, data=TSS_DISTANCE)+
  labs(x="\nDISTANCE FROM TSS (Kb)",y="FRACTION OF CONSERVED CREs (%)
\n")+
  theme_minimal()+
  theme(axis.title=element_text(size=16),
axis.text=element_text(size=20))


######## Cell types

## From TABLES2.txt, extract CREs with available DHS data on cell
types

## In R

matrix=read.table('TABLES2.txt', header = T)

matrix=na.omit(matrix$cell_types_N)

# Find quantiles
q=quantile(matrix$cell_types_N, probs = seq(0, 1, 0.1), na.rm = FALSE)

# Write quantiles in a dataframe
write.csv(as.data.frame(q), file = 'quantiles_cell_types_N.csv',
row.names = FALSE)

# PLOT conserved fraction by cell type N
cell_type = read.csv("conservation_cell_types.csv", header = T)
cell_type$N_cell_types <- factor(cell_type$N_cell_types,
levels=unique(cell_type$N_cell_types))

ggplot()+
  geom_line(aes_string(x='N_cell_types',y='Fraction_conserved'),
color='#0073ff', data=cell_type, group =1, size =2)+
  geom_point(aes_string(x='N_cell_types',y='Fraction_conserved'),
color='#0073ff',shape=19, size=5, data=cell_type)+
  labs(x="\nNUMBER OF CELL TYPES",y="FRACTION OF CONSERVED CREs (%)
\n")+
  theme_minimal()+
  theme(axis.title=element_text(size=16),
axis.text=element_text(size=20))


### Quantify correlation between number of TFBSs and degree of
conservation

# In matrix$conservation_status replace "conserved" with "0' and all
```

```
of the other states with "1"
# Then quantify correlation with a linear regression
corr = lm(conserved ~ n_cell_types, data=matrix)
summary(corr)


######## TFBS

## From TABLES2.txt, extract CREs with available DHS data on TFBS in
HepG2 cells

## In R

matrix=read.table('TABLES2.txt', header = T)
matrix=na.omit(matrix$TFBS_N)

# Find quantiles
q=quantile(matrix$TFBS_N, probs = seq(0, 1, 0.15), na.rm = FALSE)

# Write quantiles in a dataframe
write.csv(as.data.frame(q), file = 'quantiles_TFBS_N.csv', row.names =
FALSE)

# Count how many conserved and labile CREs within each quantile,
compute conserved fraction and write data into a single dataframe
'conservation_TFBS.csv'



# PLOT conserved fraction by cell type N

TFBS = read.csv("conservation_TFBS.csv", header = T)
TFBS$N_TFBSs <- factor(TFBS$N_TFBSs, levels=unique(TFBS$N_TFBSs))

ggplot()+
  geom_line(aes_string(x='N_TFBSs',y='Fraction_conserved'),
color='#0073ff', data=TFBS, group =1, size =2)+
  geom_point(aes_string(x='N_TFBSs',y='Fraction_conserved'),
color='#0073ff',shape=19, size=5, data=TFBS)+
  labs(x="\nNUMBER OF TFBS",y="FRACTION OF CONSERVED CREs (%)\n")+
  theme_minimal()+
  theme(axis.title=element_text(size=16),
axis.text=element_text(size=20))



### Quantify correlation between number of TFBSs and degree of
conservation

# In matrix$conservation_status replace "conserved" with "0' and all
```

```
of the other states with "1"
# Then quantify correlation with a linear regression
corr = lm(conservation_status ~ TFBS_N, data=matrix)
summary(corr)
```

TE analysis and replication data comparisons

In [ ]:

```
%%bash


#############################################
###SVAs analysis

## In humans, compare SVA that evolved into TEs with SVAs that did not

# From the repeat mask extract the SVAs bedfile
cat GRCh38_repeat_mask.bed | grep "SVA" > GRCh38_SVAs.bed

# Intersect the SVA bed file with all of the human peaks, including
the ones that do not have orthologs in primates, using an overlap
threshold of 25% of the length
bedtools intersect -a GRCh38_SVAs.bed -b all_human_peaks.bed  -F 0.25
-wa -wb > SVA_exapted.bed
bedtools intersect -a GRCh38_SVAs.bed -b all_human_peaks.bed  -F 0.25
-wa -wb -v > SVA_NOT_exapted.bed

# Annotate SVA_exapted.bed and SVA_NOT_exapted.bed

# Closest genes, TSS distance, strand
bedtools closest -a SVA_exapted.bed -b GRCh38_TSSs.bed >
SVA_exapted_annotated.bed
bedtools closest -a SVA_NOT_exapted.bed -b GRCh38_TSSs.bed >
SVA_NOT_exapted_annotated.bed

#Add DHS data and HepG2 TFBS data
bedtools intersect -a SVA_exapted_annotated.bed -b ENCODE_DHS_DATA.bed
> SVA_exapted_annotated_DHS.bed
bedtools intersect -a SVA_NOT_exapted_annotated.bed -b
ENCODE_DHS_DATA.bed > SVA_NOT_exapted_annotated_DHS.bed

bedtools intersect -a SVA_exapted_annotated_DHS.bed -b
ENCODE_TFBS_DATA.bed > SVA_exapted_annotated_DHS_TFBS.bed
bedtools intersect -a SVA_NOT_exapted_annotated_DHS.bed -b
ENCODE_TFBS_DATA.bed > SVA_NOT_exapted_annotated_DHS_TFBS.bed



######################################################################
###########
############# Comparison with Villar et al. (2015) data
```

```
### Replicated human peaks

# Using bedtools "window", overlap our human H3K27Ac peaks with Villar et al (2015) human H3K27Ac peaks, after liftovering their peak coordinates to GRCh38 system
# window of overlap = 1kb to find replicated H3K27Ac peaks
bedtools window -a Villar_human_H3K27Ac.bed -b our_human_H3K27Ac.bed -w 1000 > replicated_human_H3K27Ac_peaks.bed
bedtools window -a Villar_human_H3K27Ac.bed -b our_human_H3K27Ac.bed -w 1000 -v > NOT_replicated_human_H3K27Ac_peaks.bed


# Annotate replicated and not replicated peaks

# Closest genes, TSS distance, strand
bedtools closest -a replicated_human_H3K27Ac_peaks.bed -b GRCh38_TSSs.bed > replicated_human_H3K27Ac_peaks_annotated.bed
bedtools closest -a NOT_replicated_human_H3K27Ac_peaks.bed -b GRCh38_TSSs.bed > NOT_replicated_human_H3K27Ac_peaks_annotated.bed

#Add DHS data and HepG2 TFBS data
bedtools intersect -a replicated_human_H3K27Ac_peaks_annotated.bed -b ENCODE_DHS_DATA.bed > replicated_human_H3K27Ac_peaks_annotated_DHS.bed
bedtools intersect -a NOT_replicated_human_H3K27Ac_peaks_annotated.bed -b ENCODE_DHS_DATA.bed > NOT_replicated_human_H3K27Ac_peaks_annotated_DHS.bed

bedtools intersect -a replicated_human_H3K27Ac_peaks_annotated_DHS.bed -b ENCODE_TFBS_DATA.bed > replicated_human_H3K27Ac_peaks_annotated_DHS_TFBS.bed
bedtools intersect -a NOT_replicated_human_H3K27Ac_peaks_annotated_DHS.bed > NOT_replicated_human_H3K27Ac_peaks_annotated_DHS_TFBS.bed


### Opossum analysis

# After liftovering opossum peaks (Villar et al., 2015) to GRCh38 coordinates use bedtools to see how many of the 47,643 human consensus CREs having orthologs in other primates (i.e. TABLES2), also have orthologs in opossum
bedtools intersect -a TABLES2.bed -b opossum_peaks.bed -wa > human_opossum_orthologs.bed

# Count how many of of the human-opossum orthologs are primate conserved CREs, and save them in a file
cat human_opossum_orthologs.bed | grep "conserved" | wc -l
cat human_opossum_orthologs.bed | grep "conserved" > human_opossum_orthologs_conserved.txt
```
